# Amelioration of biomass and lipid in marine alga by an endophytic fungus *Piriformospora indica*

**DOI:** 10.1186/s13068-019-1516-6

**Published:** 2019-07-08

**Authors:** Vipul Swarup Bhatnagar, Prasun Bandyopadhyay, Girish H. Rajacharya, Sharanya Sarkar, Krishna Mohan Poluri, Shashi Kumar

**Affiliations:** 1 International Centre for Genetic Engineering and Biotechnology, Aruna Asaf Ali Marg, New Delhi, 110067 India; 2 Department of Biotechnology & Centre for Transportation Systems (CTRANS), Indian Institute of Technology Roorkee, Roorkee, Uttarakhand 247667 India

**Keywords:** Commensalism, Metabolomics, Endophytic fungus, Biofuel, Co-culture

## Abstract

**Background:**

Many studies have been carried out on the growth-modulating efficiency of plants by the colonization of an endophytic fungus *Piriformospora indica.* However, studies involving the co-culture of alga with endophytic fungal strains for enhanced biodiesel production are rare. In this study, the interaction between *P. indica* and *Parachlorella kessleri*-I, a marine algal strain, was assessed at metabolic level.

**Results:**

In association with an endophytic fungus, the algal biomass enhanced from 471.6 to 704 mg/L, and the fatty acid methyl ester (FAME) profile of *P. kessleri*-I increased substantially. In case of FAME profile of co-cultured *P. kessleri*-I, two essential components of biodiesel, i.e. elaidic acid and oleic acid, increased by 1.4- and 1.8-fold, respectively. To ascertain changes in the metabolic profile of *P. kessleri*-I by *P. indica* co-culture, gas chromatography–mass spectrometry (GC–MS)-based untargeted metabolomics study was performed to identify the metabolites involved; and differential nature of the essential metabolites was also confirmed using HPLC and LC–MS. Significant modulation of the bioactive metabolites such as succinate, oxo-propanoate, l-alanine, glutamate, acetate and 1,2 propanediol, hydroxy butane was observed.

**Conclusion:**

The metabolites like glutamate and succinate that usually belong to the GABA shunt pathway were observed to be upregulated. The pathway links nitrogen metabolism and carbon metabolism, thus influencing the growth and lipid profile of the algae. These differential metabolites thus indicated the important commensal association between the endophytic fungus and autotrophic marine alga, and established that endophytic fungus can be handy for the sustainability of algal biofuel industries.

**Electronic supplementary material:**

The online version of this article (10.1186/s13068-019-1516-6) contains supplementary material, which is available to authorized users.

## Introduction

Algae-based biodiesel is a new energy source that has been explored worldwide as the third-generation biofuel. The oil content in algae can be readily converted to petroleum-like products for serving the drop-in replacements for gasoline, diesel, jet fuel, etc. [1]. Moreover, compared to diesel, petroleum and other sources of fossil fuels, the combustion of algal biofuel does not produce any sulfur oxide or nitrous oxide. It generates reduced quantity of carbon monoxide and unburned hydrocarbons. Moreover, the associated greenhouse gases (GHG) footprint is way lesser than the other conventional fossil fuels [2]. Algae-based fuel yields considerably more energy per unit area and can be cultivated easily in the land that is unsuitable for agriculture [3]. However, this technology is not yet established completely for commercial-scale production. At present, enhancement of biomass and fatty acid methyl ester (FAME) content is also a major target for biofuel industries apart from harvesting and lipid extraction [4–7].

Currently, most of the studies have focused on the use of fungal pellets formed in the suspension medium for harvesting algal cells. These fungal pellets or para-globules are formed due to the varying morphology of fungal strains. The variation in morphology can be of different types, for instance, microscopic aggregates, dispersed hyphae, clump formation, pellet formation or denser spherical aggregates. Pelletization due to filamentous fungi can be of various types, namely fluffy pellets, smooth-compact pellets or smooth-hollow pellets. The formation of pellets usually takes place either from one spore, termed as a non-coagulation pellet or from the aggregation of spores at an early stage, which is termed as coagulation pellet [8, 9]. Few studies, that have been carried out for depicting harvesting efficiency of fungal pellets, suggest that algal cells have a net negative surface charge due to the presence of carboxylic, phosphor diester, phosphoric, hydroxyl and amine groups. It may be neutralized by the surface charge of mycelia and hyphae of fungal strains that contain positively charged polysaccharides [10]. This, in turn, might enable physical interaction between the two completely different organisms, thus paving the way toward the understanding of their metabolic profile changes [11].

The most common example of algal and fungal interaction of two complicated populations in nature is lichen, which is the symbiotic association of two or more than two species [10, 12]. Both autotroph–heterotroph symbionts thrive by carrying out various functions and by regulating their metabolic pathways. The symbiotic interaction of endophytic fungi with marine microalgae resulted in a bioactive metabolite that has the potential to act as an active pharmaceutical ingredient [13]. However, the modulating effect of endophytic fungal strain on microalgae has not yet been reported.

*Piriformospora indica* is a well-studied basidiomycete endophytic fungus that can be grown axenically, thus making it the most suitable among endophytic fungal species for studying the mechanism and evolution of mutualistic symbiosis [14]. It colonizes the monocotyledonous and dicotyledonous plants easily and stimulates the growth of many plants [15–17]. *P. indica* helps in mobilizing the insoluble phosphate in an energy-dependent process and translocates phosphate into the host, thereby mimicking the mutualistic relation between two species [14]. The fungus receives carbohydrate and growth factors from the plant while it helps plants by improving its growth-enhancing functions, including increased nutrient absorption. Studies deciphering the fungal genome have revealed that *P. indica* depicts the missing relation between saprophytic and obligate biotrophic mutualistic fungal species. Furthermore, few other studies on *P. indica* focus on the co-existence of characteristics of both symbiotic and obligate biotrophic fungi, which is attributed to the presence of genes responsible for biotrophic lifestyle and the absence of genes for nitrogen metabolism [14, 18].

The current study is designed to elucidate the effect of *P. indica* on *Parachlorella kessleri*-I (*P. kessleri*-I) algal biomass and lipid profile under a co-cultivation environment. Further, to observe the variation in the metabolic profile of algal cells under the influence of *P. indica*, gas chromatography–mass spectrometry (GC–MS)-based untargeted metabolomics studies were conducted. High-pressure liquid chromatography (HPLC) and liquid chromatography–mass spectrometry (LC–MS) were used to substantiate the differential presence of the metabolites identified by GC–MS. The results evidenced for enhanced lipid accumulation profile of the algal cells under the biotic stress of *P. indica*. Thus, the endophytic fungus may serve as an important symbiont for the algal biofuel industries.

## Materials and methods

The marine micro algal *P. kessleri*-I cells were previously isolated in our lab and maintained in Tris–Acetate Phosphate (TAP) medium [19]. *P. kessleri*-I cells were inoculated with a dilution of 3 × 10^5^ cells/mL in 250-mL flasks in triplicates for each set of TAP medium with working volume of 150 mL and then incubated at 30 °C, 150 rpm under photosynthetic light of 179.8 µmol/s/m^2^ intensity for 12 days. The fungal strain—*P. indica*—was a kind gift from Dr. Narendra Tuteja, ICGEB, New Delhi and it was maintained on Hill and Kaefer medium [20]. To prepare the fungal inoculums, chlamydospores were harvested with 0.02% (v/v) Tween-20 aqueous solution from 7-day-old incubated *P. indic*a culture and washed thrice with autoclaved water. Spores were then inoculated in Hill and Kaefer Petri dishes and incubated for 7 days at 30 °C for sporulation [21]. After 7 days of incubation, spores were harvested from the Petri dishes with 10 mL of autoclaved Milli-Q water and filtered through Mira cloth of 20–25 µm pore size, supplied by Calbio chem. Then, these harvested chlamydospores of *P. indica* were inoculated at a dilution of 2.85 × 10^5^ spores/mL (approximately), for pellet formation in Hill and Kaefer medium. After 7 days of incubation, pellets were harvested and washed with autoclaved Milli-Q and co-inoculated with *P. kessleri*-I cultures in Tris–Phosphate medium. These co-cultures along with pure cultures of *P. kessleri*-I and *P. indica* as controls were incubated at 30 °C, 150 rpm with 179.8 µmol/s/m^2^ light intensity for 12 days respectively in Tris–Phosphate (TP) medium.

### Observation by light microscope

*Parachlorella kessleri*-I and *P. indica* conjugated pellets were harvested and dipped in Lactophenol Cotton Blue dye to stain chitin present in hyphal cell wall of *P. indica*. The mesh network was observed under 60× and 100× magnifications under upright Nikon Eclipse Ni microscope (Nikon Corporation, Japan).

### Estimation of growth by chlorophyll content and dry cell weight

The controls and co-cultures of *P. kessleri*-I and *P. indica* were incubated for 12 days. Total chlorophyll content and dry cell weight were estimated at regular time intervals on the 3rd, 6th, 9th and 12th day. Estimation of total chlorophyll content was done using the method described by Porra et al. [22]. Extraction of total chlorophyll from algal–fungal mesh was done by taking 1 mL of the macerated culture in 2 mL Eppendorf tubes from each of the setups and then centrifuging them at 5000 rpm for 10 min. The supernatant was discarded and the pellets were washed with Milli-Q water and re-suspended in 1 mL methanol with gentle tapping. Then, the extracts were incubated in dark for 30 min and centrifuged at 5000 rpm at 20 °C. Then from each of the extracts, 200 μL of the upper methanol layer was taken in 96-well EIA/RIA Corning plate (Corning Incorporation, USA) for absorbance measurements at 665 and 652 nm wavelengths simultaneously in Multimode micro-plate reader Spectramax M3 (Molecular Devices, USA).

Chlorophyll content was calculated using the following model equations [22]:

Chl a: 18.22A^665^ – 9.55A^652^

Chl b: 33.75A^652^ – 14.96A^665^

Chla+b: 24.23A^652^ + 3.26A^665^

Dry cell weight was estimated after harvesting co-cultures and controls at 5000 rpm, 20 °C on the 3rd, 6th, 9th and 12th day. Then, the harvested biomass of each experimental set was dried at 60 °C in hot air oven for 8 h and their respective weights were recorded.

### Estimation of lipid accumulation by fluorescence

Lipid accumulation in the co-culture and pure culture of *P. kessleri*-I was estimated at different stages by Nile Red-based neutral lipid fluorescence intensity. Quantification of neutral lipid was performed in 96-well costar plate in triplicates. At each time interval (on 3rd, 6th, 9th and 12th day), 1 mL of culture was taken from each set and centrifuged at 5000 rpm, 20 °C for 10 min. Then, the supernatant was discarded and the pellets were re-suspended in 1 mL Milli-Q water. About 150 µL was mixed with 20 µL of Nile Red containing 25% DMSO solution (6 µg/mL). Simultaneously, 150 µL of algal culture was mixed with 20 µL of 25% DMSO with no Nile Red as a control. The control unstained cells were incubated at 40 °C, 150 rpm for 10 min. Then, the fluorescence was recorded using Spectra max spectra-fluorometer at excitation wavelength of 485 nm and emission wavelength of 552 nm [23]. Fold increase of the lipid content is calculated as follows:

$$\frac{{{\text{Flu}} . {\text{intensity of stained cells}} - {\text{Flu}} . {\text{intensity of unstained cells}}}}{{{\text{Optical density of the unstained cells at }}750\;{\text{nm}}}}$$

### Lipid extraction and estimation of fatty acid methyl ester profile

Total lipid was extracted as reported [24]. After 12 days of incubation, the wet biomass is harvested by centrifuging at 5000 rpm and dried at 60 °C. Around 1 g of dry biomass of each experimental set was crushed using mortar and pestle, and the crushed biomass was mixed with 1 mL of chloroform along with 2 mL of methanol, which was then kept for shaking at 150 rpm for 12 h. The mixtures were again shaken at 150 rpm for 1 h after adding 1.5 mL of distilled water, to separate the mixture into bi-phasic layers. From this bi-phasic layer, the bottom layer of chloroform was separated and filtered out using Whattmann filter paper which was then evaporated by Nitrogen flushing. Transesterification of neutral lipid was done using 2 M methanolic-KOH, added in the ratio of 200 µL per 20 mg of total lipid. Heptadecanoic acid methyl ester was added as an internal standard at a concentration of 200 µg/mL to each of the samples for quantification of the fatty acid methyl ester (FAME) content. Transesterified neutral lipids were extracted by 1 mL *n*-Hexane (HPLC grade) for performing GC–MS. For quantification of FAME, Agilent 7890A series GC system (Agilent Technologies; Singapore) comprising an Omega Wax 250 column (30 m × 0.25 mm 0.25 µm) coupled with an Agilent 7000 QQQ MS was used. All essential parameters for GC–MS experiments are included in Additional file 1: Methods section.

### UATR-FTIR measurement of cell–cell interaction

The co-culture and pure culture biomasses were harvested at 5000 rpm after complete growth. The samples were collected and washed with Milli-Q water and centrifuged. The pellets were then freeze dried and stored for further analysis by Mid Infrared range (MIR range) Perkin Elmer’s MIR/NIR Frontier DTGC/KBr UATR (Perkin Elmer, Singapore) with built-in diamond universal attenuated total reflectance with wave number ranging from 600 to 4000 cm^−1^. Total 100 scans were averaged for each sample and background noise with spectral contribution of water from paste was subtracted from Universal Attenuated Transform Reflectance-Fourier Transform Infrared (UATR-FTIR) spectrum of individual samples [25, 26].

### Metabolite extraction and GC–MS-based untargeted metabolite detection

The metabolites of the samples were extracted using the protocol reported by Villas-Boas et al. [27]. About 500 mg of wet biomass for 18 replicates of both co-culture and controls was collected and then quenched overnight at − 20 °C in a solution containing glycerol and wate0r in a ratio of 5:1 with 13.5 gm/L of sodium chloride added in it. Then, pellet from each replicate was washed twice with a 1:1 solution of glycerol and water. Pellets were then suspended in super-cooled methanol and vortexed for 5 min and then methanol was evaporated using nitrogen flushing. Metabolites for each sample were concentrated by repeating the methanol extraction step for three times. Derivatization was carried out using 100 µL of BSTFA (*N*,*O*-Bis(trimethylsilyl)trifluoroacetamide) mixed with 1% TMCS (Trimethylchlorosilane) of Cerilliant (Sigma Aldrich) followed by incubation at 80 °C on a hot plate for approximately 30 min. 100 µL of derivatized extracts of each sample with internal standard Heptadecanoic methyl ester was used for GC–MS-based analysis.

GC–MS equipped with an HP-5 column (30 m * 0.25 mm ID, 0.25 μm thickness, Varian) coupled with an Agilent 7000 QQQ MS was used for the analyses of metabolic profiles of the samples. Electron ionization system with ionization energy of 70 eV was used and 99.99% pure helium was used as a carrier gas at a constant flow rate of 1.1 mL/min. Mass transfer line and injector temperature were set at 220 °C and 250 °C, respectively, and the oven temperature was programmed at 60 °C for 1 min, then increased at the rate of 5 °C/min to 180 °C for 1 min, then again increased at 10 °C/min to finally 310 °C for 2 min. 1 µL of sample from each set was injected in the split mode 5:1 during analysis. The signals were recorded in full-scan mode (*m*/*z* 20–600, 250 scans/ms).

GC–MS raw data were obtained and deconvolution and metabolite identification of this complex data were done by comparing their mass spectra with those obtained from authentic samples and/or the NIST (National Institute of Standards and Technology) mass spectral database using AMDIS (Automated Mass spectral Deconvolution and Identification System) and MassHunter software. This whole process was repeated at least for 18 biological replicates. Once the data were preprocessed using AMDIS software, the metabolites were screened for bioactive compounds using KEGG (Kyoto Encyclopedia of Genes and Genomes) compound portal of KEGG database. Relative abundance percentages of these bioactive metabolites were calculated using AMDIS software averaged manually and then plotted in the clustered heatmap. The heatmap was generated using BioConductor’s R script package gplots combined with R ColorBrewer’s palettes (Warnes et al. [28]).

### Metabolite extraction and liquid chromatography–mass spectrometry (LC–MS)-based untargeted metabolite detection

Samples were grown for 12 days and harvested at 5000 rpm (2348×*g*) 4 °C for 10 min using Eppendorf centrifuge 5424R (Eppendorf, Germany). Pellets were rinsed with Milli-Q water and ~ 500 mg of each was re-suspended in 0.5 mL of Methanol:water (80:20) solvent and kept in − 80 °C overnight. After quenching, samples were homogenized by a sonicator (Sonics-VCX 500, USA) using 30% pulse for 15 min. Samples were centrifuged and passed through 0.45-µm filter. 100 µg/mL no-valine was used as an internal standard along with samples for LC–MS–MS analysis.

Orbitrap Fusion Lumos Ttribrid Mass Spectrometer equipped with column Acclaim Trinity P2 (100 × 2.1 mm, 3 µm) (Thermo Fischer Scientific, Singapore) was used in mix mode for metabolic analysis. Essential parameters for buffer system and LC–MS–MS are mentioned in Additional file 1: Table S1. The xcalibur software was used for the LC–MS–MS data acquisition (Thermo Scientific Version 3.0) and exported in RAW format. After data acquisition, each RAW file was imported in Compound Discoverer 3.0 (Thermo Scientific) and non-nested grouping was done with 7 replicates. Each sample group, i.e. algal, fungal, and co-culture were then assigned with ratio type (co-culture/alga; co-culture/fungal) for depicting *p* value. Untargeted metabolomic analysis with peak filtering compound annotation by online databases was selected as the workflow template as shown in Additional file related to workflow. Multivariate analysis was performed using the Compound Discoverer 3.0 (Thermo Scientific).

### High-pressure liquid chromatography (HPLC)-based analysis of cell extracts

After 12 days of incubation, samples were harvested at 5000 rpm 25 °C using Eppendorf centrifuge (Eppendorf, Germany). Then pellets were washed and re-suspended in 1 mL of Milli-Q water and then sonicated at 30% pulse for 10 min with Sonics-VCX 500 sonicator (USA). Cell extract was collected and filtered using a 0.45-µm syringe filter. We analyzed the concentration of hydroxy-glutamate and succinate in cell extracts using high-pressure liquid chromatography equipped with Aminex HPX-87H column. Derivatization of glutamate to form hydroxy-glutamate was carried out in 2-mL microcentrifuge tubes at 45 °C for 90 min. Samples were mixed with 0.2 mL of 1 M KNO_3_, and reaction was started by adding 0.04 mL of 12 M HCl and stopped by adding 0.2 mL 2 M NaOH. Quantification of derivatized glutamate was studied using an Aminex HPX-87H column (300 × 7.8 mm, 10 µ) Bio-Rad, USA; Ion Exchange column and RI detector. HPLC parameters used for glutamate and succinate study are mentioned in Additional file 1: Methods section.

### Metabolic pathway analysis (MetPA)

Uncommon compounds identified in co-culture of *P. indica* and *P. kessleri*-I with respect to their controls were searched by their KEGG IDs using KEGG database [29, 30]. The KEGG IDs of these compounds were then used in Metabopathway (MetPa) analysis web interface for positioning them in global metabolic pathways. Compound hits obtained from MetPA were plotted in the stacked bar graph against total metabolites present in respective pathways of the KEGG database with *Chlamydomonas reinhardtii* taken as a model organism [31].

### Statistical data analysis

Each statistical datum was calculated from biological triplicates of each experimental set and error line was calculated by standard deviation between each set plotted using GraphpadPrism version 5. For all the statistical analyses, ANOVA test was used.

## Results

### Co-culturing of *P. kessleri*-I with *P. indica*

**Fig. 1:**
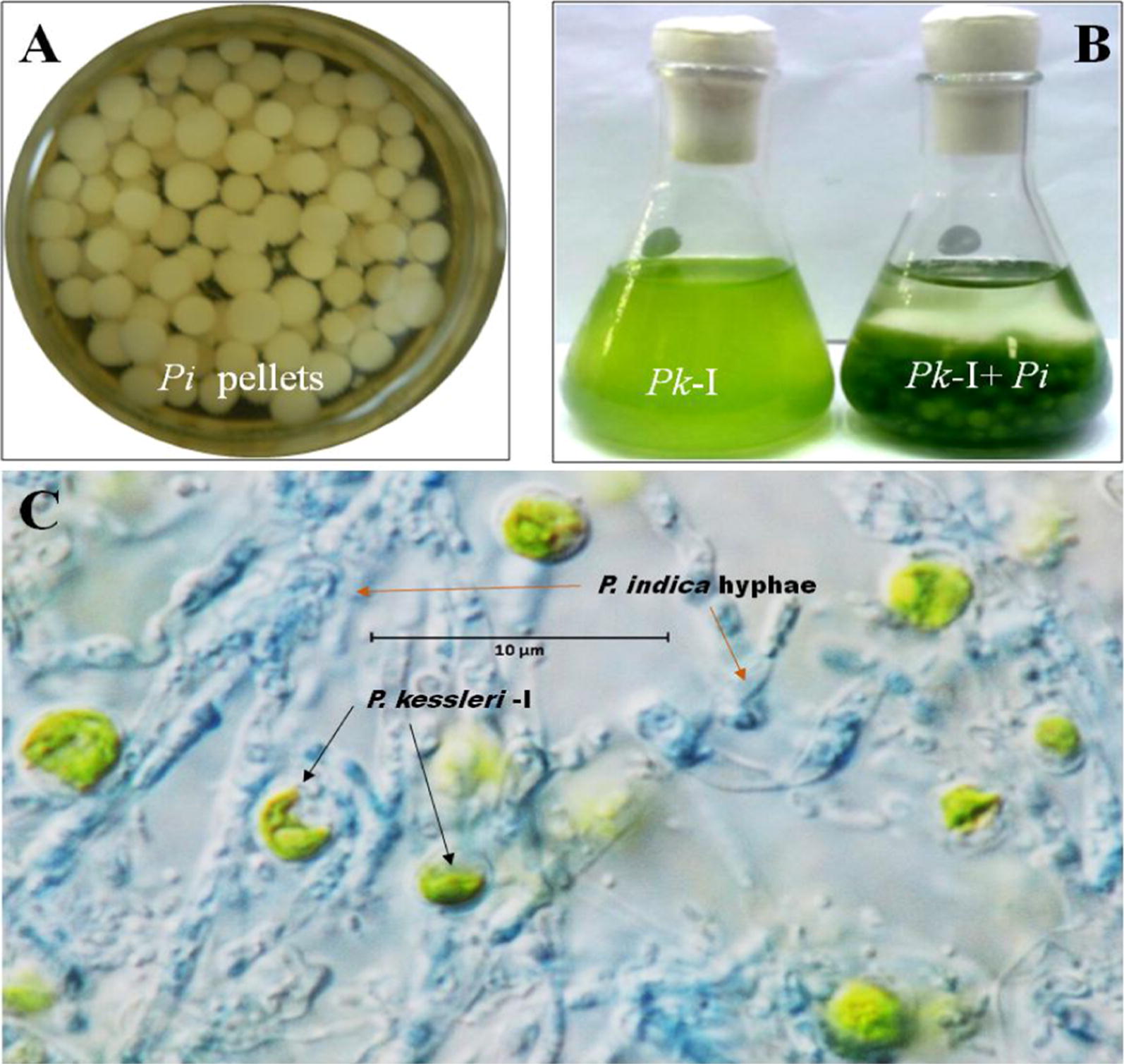
**A**–**C** Culture of endophytic fungus *Piriformospora indica* (*Pi*) and para-globules formation in Hill and Kaefer medium for studying fungal interaction with marine alga *Parachlorella kessleri*-I (*Pk*-I). **A** Spore formation of *Pi* on Hill and Kaefer medium after 7 days. Then, *Pi* spores (2.8 × 10^5^ spores/mL) were subjected to form fungal pellets. **B** After 7 days of *Pi* pellet formation, they were co-cultured with *Pk*-I on 0th day culture and incubated for 12 days. **C** Interaction of *Pi* and *Pk*-I studies under Nikon Ni light microscope (100× magnification) after 12th day. *Pk*-I cells (green) interacting with *Pi* hyphae (stained with cotton blue dye). The interacting *Pk*-I cells are shown with black arrows and *Pi* hyphae are indicated by red arrows

Using the co-culture of *P. kessleri*-I with *P. indica*, changes in growth, lipid content, UATR-based physical interaction and untargeted metabolic profile were studied. Tris–Phosphate (TP), a standard maintenance medium, was used to co-culture *P. indica* pellets with *P. kessleri*-I along with their pure cultures as controls (Fig. 1A, B). We observed there was no change in the growth of *P. indica* pellets in TP medium, due to the absence of peptone and carbon contents that are important for spore formation. Under Nikon Ni light microscope at 100× magnification, we observed hyphal networks of *P. indica* interacting with *P. kessleri*-I cells (Fig. 1C). Formation of a biofilm of fungal hyphae with algal cells entrapped in it resulted in their physical interaction. It was essential to validate that there is cell–cell interaction between the two species. For this, Universal Attenuated Fourier Transform Infrared Spectroscopy (UATR) was used to determine the cell surface functional groups responsible for adhesion of the two different cells. In the case of co-cultures, we observed higher peak intensities of C–O bond vibration of carbohydrate (Additional file 1: Figs. S1–S3, Tables S2–S4) [25]. This UATR-based observation suggested the strong possibility for physical interaction-based commensalism between the two species [25, 32].

### Estimation of growth by correlation ratio of Chlorophyll content and dry cell weight

**Fig. 2:**
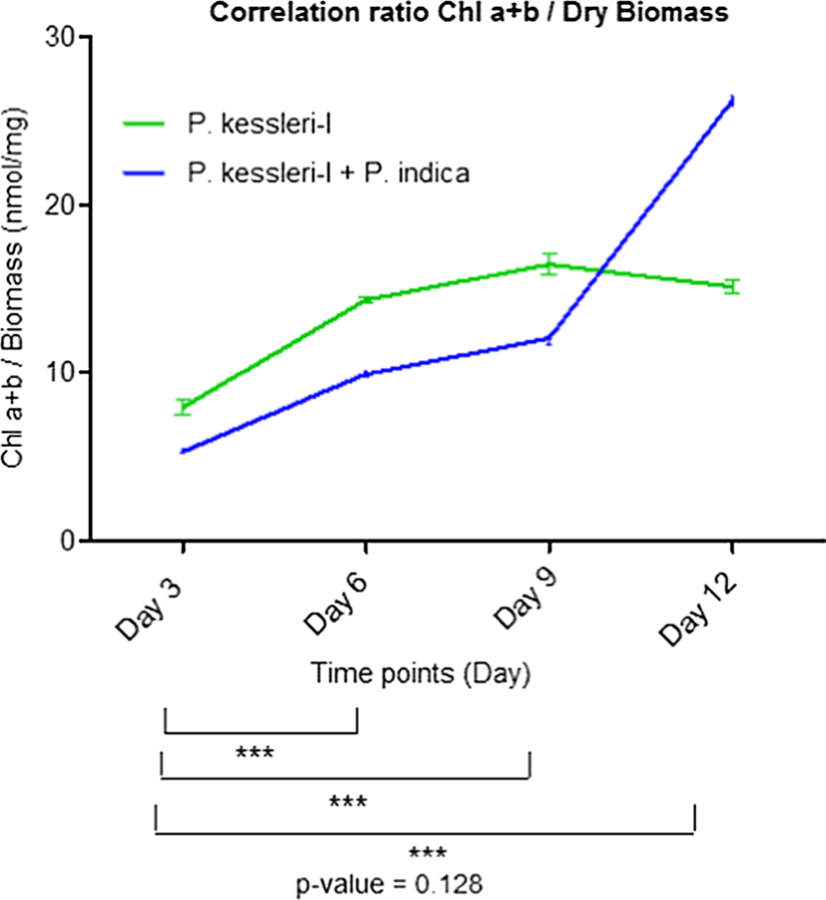
Estimation of correlation ratio of chlorophyll content to biomass of co-cultured *P. kessleri*-I (*Pk*-I) with respect to pure culture. To assess the effective enhancement in growth yield under the influence of biotic stress given by *P. indica*, dry correlation ratio of chlorophyll content to biomass of *P. kessleri*-I at pH 7 was analyzed at each time point. There was about twofold increase with respect to *P. kessleri*-I control

We have analyzed the total chlorophyll content for co-culture and the control in nmol/mL. According to previously published studies, the increase in biomass is relevantly equivalent to the increase in total chlorophyll content (nmol/mL) [33, 34]. We observed that there was a substantial increase in the total chlorophyll content of *P. kessleri*-I in co-culture, i.e. approximately 11.7 nmol/mL higher than the biomass of pure culture (Table 1). Then, the correlation ratio of total chlorophyll content and their consecutive dry cell weight at different time points (3rd, 6th, 9th and 12th day) were plotted (Fig. 2). Under the association of an endophytic fungus, the algal biomass was enhanced by 1.5-fold (from 471.6 to 704 mg/L) on the 12th day (Table 2). We have also validated the correlation ratio data with standard curve of total chlorophyll content vs. dry biomass of pure culture (Additional file 1: Fig. S4).

**Table 1:** Total chlorophyll content (average) estimation of co-cultured and pure culture of *P. kessleri*-I at different time points

Time point	3rd day	6th day	9th day	12th day
*P. kessleri*-I (nmol/mL)	2.06365	3.9715	5.2109	7.13305
*P. kessleri*-I + *P. Indica* (nmol/mL)	2.7694	5.67105	7.41755	18.455

**Table 2:** Dry cell weight (average) estimation of co-cultured and pure culture of *P. kessleri*-I and *P. indica* at different time points

Time point	3rd day	6th day	9th day	12th day
*P. kessleri*-I (mg/L)	260 ± 10	276.6 ± 5.7	316.6 ± 10.59	471.6 ± 9.6
*P. indica* (mg/L)	152 ± 7.2	169 ± 11.53	160.6 ± 7.09	164 ± 16
*P. kessleri*-I + *P. indica* (mg/L)	520 ± 10	570 ± 10	619 ± 14.17	704.4 ± 6.02

### Estimation of modulation in FAME profile

**Fig. 3:**
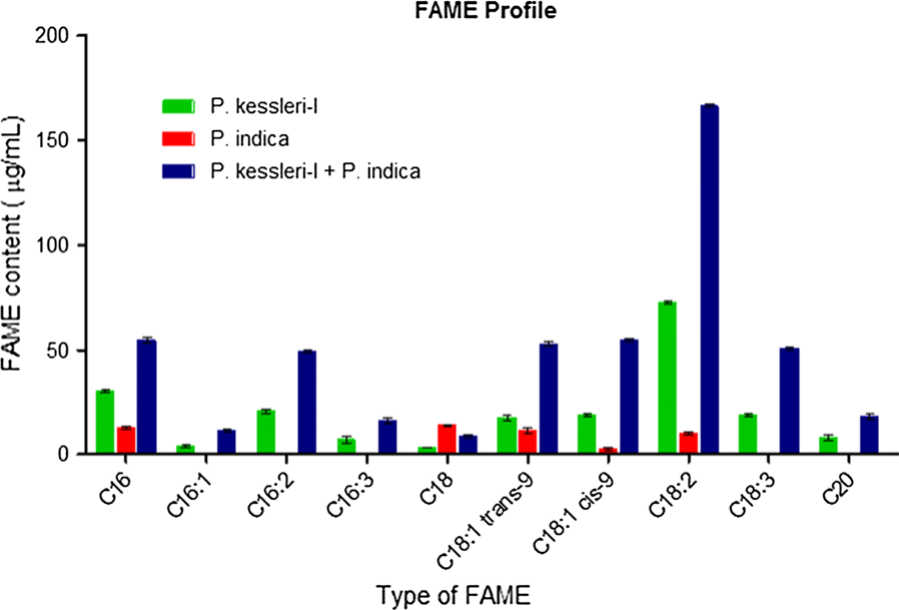
Estimation of FAME content of *P. kessleri*-I co-cultured with *P. indica*after 12 days. It was found that there was modulation in fatty acid methyl ester content of *P. kessleri*-I under the commensal effect of *P. indica.* Approximately onefold enhancement in the FAME profile of *P. kessleri*-I was observed under the influence of *P. indica*

We have measured the changes in the neutral lipid content at different time points for each experiment set up by Nile Red fluorescence normalized intensity. We observed maximum fluorescence intensity in co-cultured algal cells between day 6 and day 12, whereas in algal controls the fluorescence intensity decreased on the 12th day (Table 3), may be due to the cessation of lipid biosynthesis. We observed about onefold increase in Nile Red fluorescence intensity for co-culture on day 12. Increase in neutral lipid fluorescence intensity of co-cultured algae has motivated us to analyze the GC–MS-based FAME profile, which reflects the changes in the neutral lipid content. The oleic and elaidic acid contents were observed to increase drastically by 1.4- to 1.8-fold in the FAME profile of co-cultures (Fig. 3). Both of them are essential for improving the quality of biodiesel. Internal standard heptadecanoic acid methyl ester was used for correcting system error; its chromatogram has been shown in Additional file 1: Fig. S5.

**Table 3:** Neutral lipid normalized Nile Red fluorescence intensity at different time points

Time point	Day 3	Day 6	Day 12
Control	15.81 ± 5.28	23.89 ± 10.142	20.93 ± 17.402
*Pi*	6.27 ± 12.02	5.68 ± 14.732	5.98 ± 1.37
*Pk*+*Pi*	20.48 ± 9.105	26.20 ± 5.418	29.42 ± 13.729

### Gas chromatography–mass spectrometry (GC–MS)-based untargeted metabolomic analysis

**Fig. 4:**
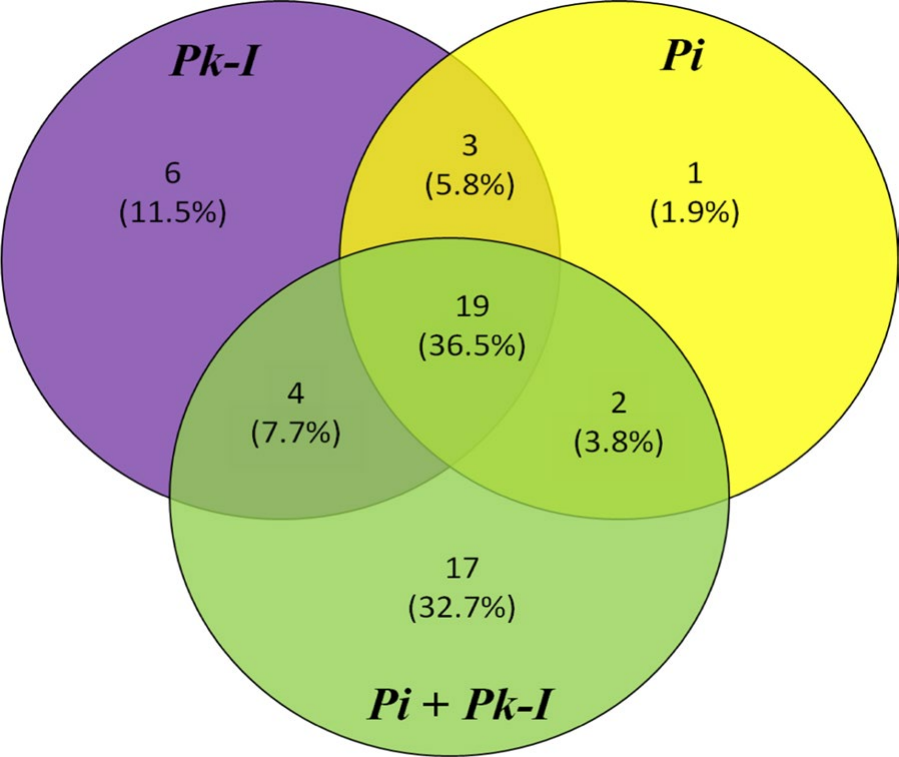
Venn diagram was drawn of metabolomic analysis of *P. indica*–*P. kessleri*-I co-culture after 12 days of incubation. The number of bioactive metabolites from *P. indica* and *P. kessleri*-I during co-culture as well as in an individual cultures is shown using Venn diagram

**Fig. 5:**
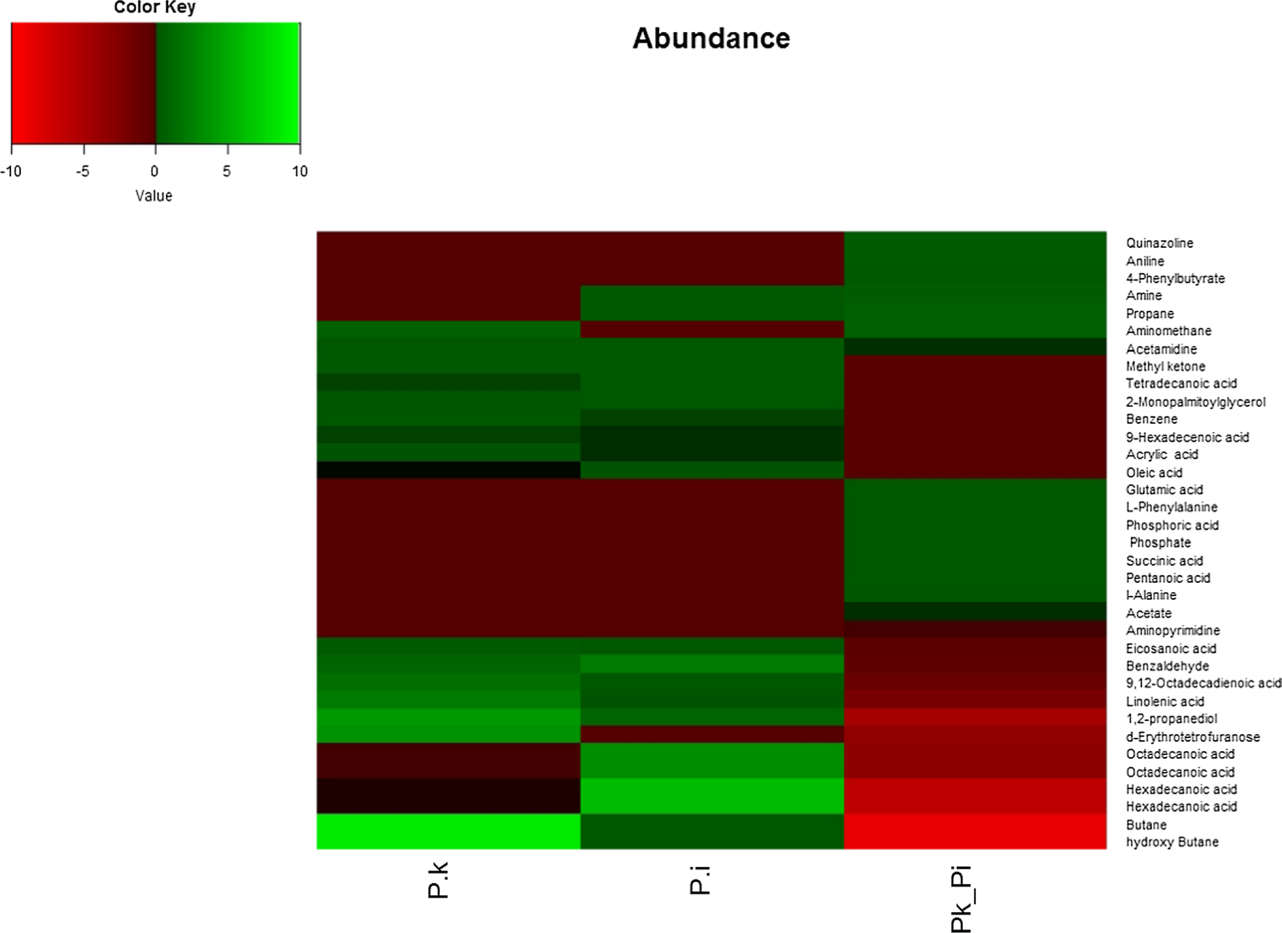
Abundance of relative % values of upregulated and downregulated bioactive metabolites in *P. kessleri*-I–*P. indica* co-culture. Bioactive compounds screened from co-cultured metabolome are depicted in heatmap with respect to the Abundance value of *P. kessleri*-I and *P. indica* controls. Ranges of colors are specified as follows: − 10 to − 0.1 as Red, 0.09 to 0.1 as Black and 0.51 to 10 as Green. Heatmap was generated using gplots of R script. Heatmap was used to visualize the comparative relative increase in the abundance value of induced metabolites with respect to *Pk*-I and *Pi* controls thus making it easy for visualizing the comparison

**Fig. 6:**
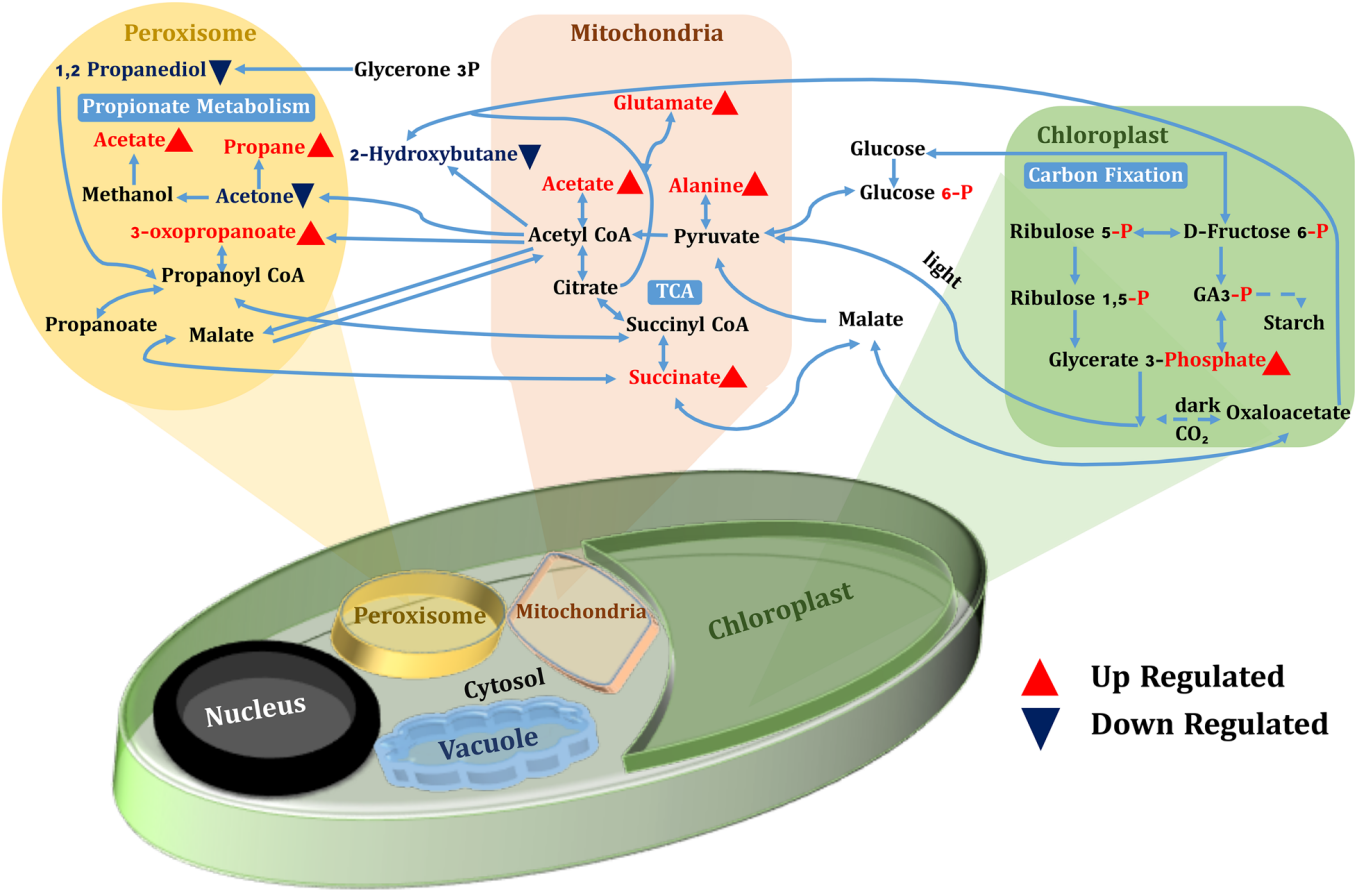
Carbon metabolism internetworking; in the figure, red-colored metabolites are the induced metabolites and blue=colored ones are the repressed metabolites

After assessing the modulation in growth and neutral lipid content of *P. kessleri*-I by co-culturing with *P. indica*, it was also essential to decipher the changes that were taking place at the metabolic level. To identify induced and repressed metabolites in co-culture, we have generated three metabolic libraries as follows: two control metabolic libraries each from pure cultures of alga, fungus and one library from co-culture. Pre-processing and manual screening of GC–MS data gave a total of 366 hits, among which 152 active metabolites were detected. We observed about 57 metabolites with 19 bioactive metabolites after manual screening through KEGG database with *Chlamydomonas reinhardtii* taken as the model organism. Among these 57 bioactive compounds, 42 were identified in co-culture, 32 in *P. kessleri*-I control and 25 in *P. indica* control (Table 4). Venn diagram plotted using Venny 2.1.0 shows the set of compounds common or uncommon in the case of co-culture and their controls (Fig. 4) [35]. PCA score plot from XCMS database was used to determine the relationship between samples and utilized the loading plot to observe the correlation between the metabolites and the sample types. We have observed the clustering of dots in PCA-2D plot due to divergence in the metabolite and sample type [36]. Each dot in PCA-2D, score plot and loading plot denotes a particular metabolite. They belong to different experimental sets and the clustering is done based on the sample types (Additional file 1: Fig. S6). A major number of metabolites, with high probability of their occurrence, were clustered in co-culture and algal control groups. We observed about 60% variance in the distribution of metabolites between different sample groups. Also, this interactive PCA-based clustering of metabolite hits in their sample types depicted the difference in the profile of the co-culture metabolic library (Additional file 1: Fig. S7). Further, to determine the relative hierarchical cluster analysis, we have generated the heatmap using abundance percentage (%) values of bioactive metabolites. In our untargeted approach, we have subtracted the abundance percentage of identified bioactive compounds detected in co-culture from those of the controls. After subtracting the abundance percentage of co-culture metabolites from the combined sum of their abundance in the controls, the higher ones were being considered to be induced. Interestingly in our study, metabolites such as succinate, oxo-propanoate, l-alanine, glutamate, and acetate were induced in co-culture. These metabolites were depicted in heatmap using R studio-based gplots (Fig. 5), and also in carbon internetworking pathway mapping (Fig. 6). The repressed metabolites with a notable decrease in relative abundance such as 1,2 propanediol, hydroxy butane, acetone and the abundance percentage values of all the metabolites are presented in Additional file 1: Table S5.

**Table 4:** Bioactive compounds identified after AMDIS-based pre-processing

	Total metabolites detected	Functional metabolites screened
*P. kessleri*-I	177	32
*P. indica*	85	25
*P. kessleri*-I + *P. indica*	110	42

### Classification of metabolite hits in global metabolic pathways

**Fig. 7:**
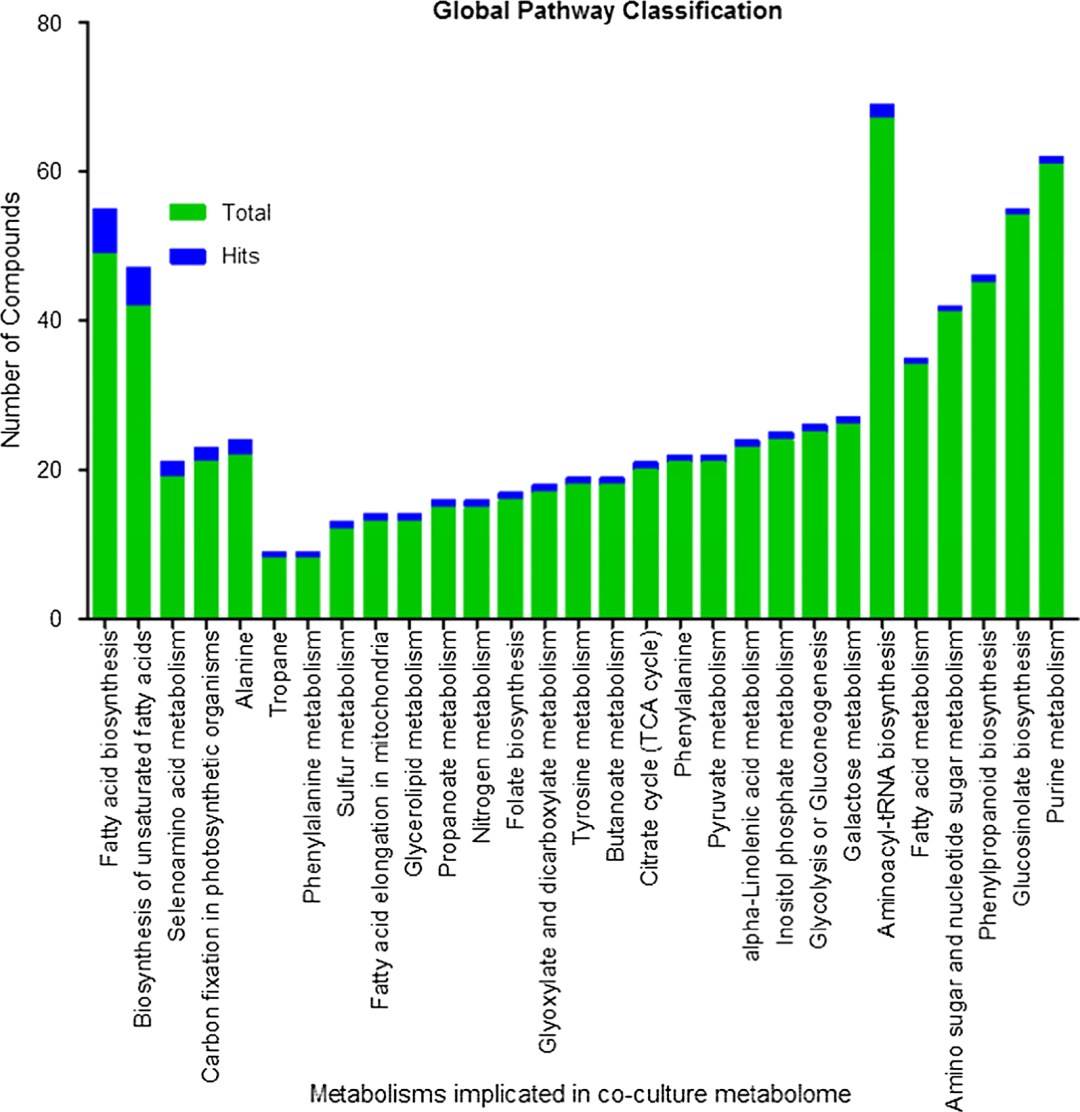
Global metabolic pathway classification of metabolomics data of *P. indica*–*P. kessleri*-I co-culture. Total number of bioactive metabolites identified in co-culture of *Pi* and *Pk*-I (*P. indica* and *P. kessleri*-I), positioned in global metabolic pathways from KEGG database using MetPA online tool of MetaboAnalyst. The bioactive metabolites of co-cultured *Pk*-I–*Pi* are classified according to their biological functions

**Fig. 8:**
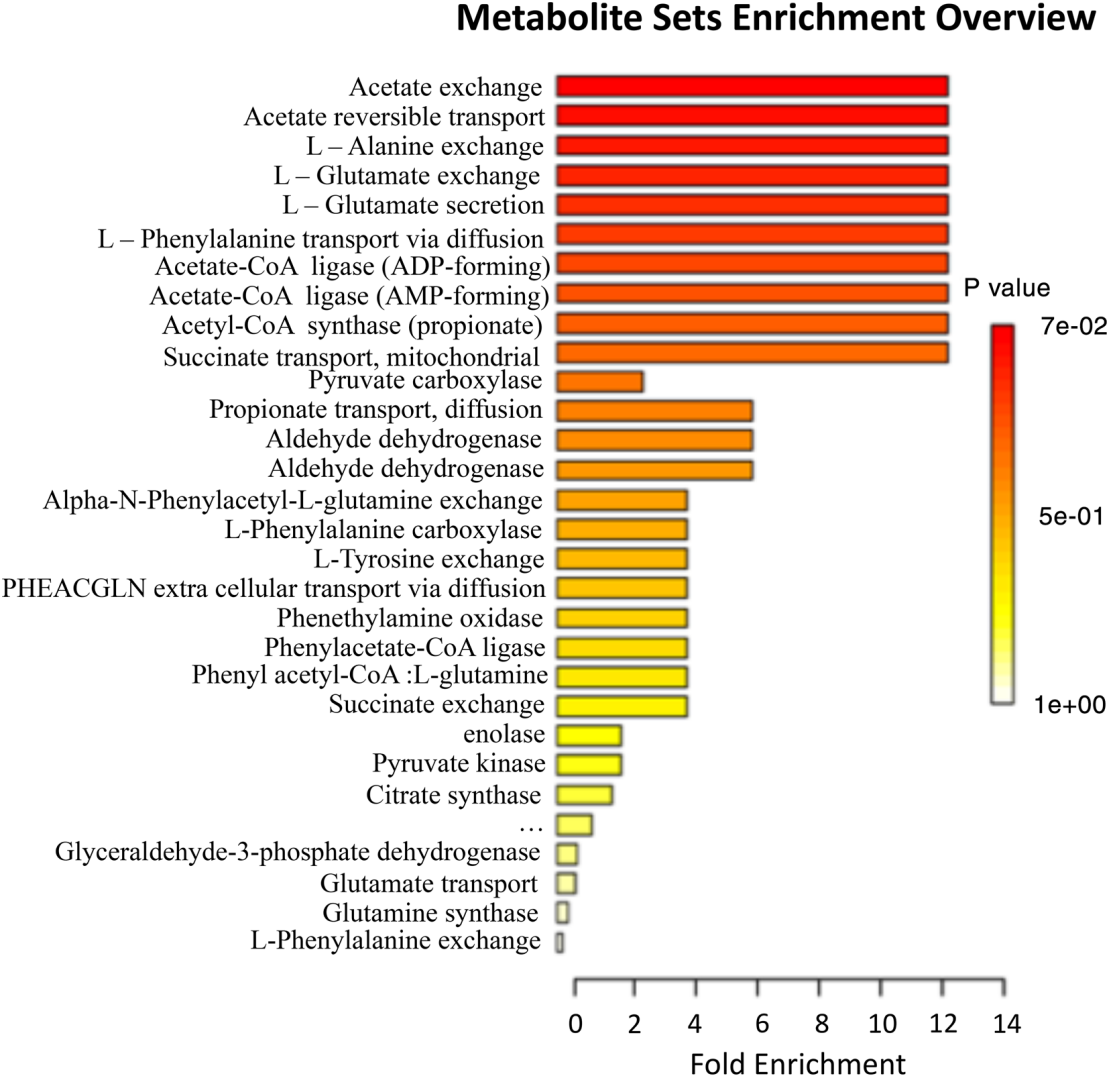
Enrichment analysis of co-culture metabolites classified in global pathways using *p* value and fold enrichment pathways as classified in Table 5. Carbon metabolism and glutamate linked pathways showed increased *p* value with a significant increase in their fold change in the co-culture metabolite hits

The induced metabolites were categorized into their cognate metabolic pathways by MetPa online tool using their KEGG IDs. Metabolites classified in global metabolic pathways were plotted in a stacked bar graph (Fig. 7) and their FDR values and *p* values are shown in Fig. 8 and Table 5. Most of the induced metabolites belong to carbon metabolism, fatty acid biosynthesis and amino acid metabolism. The observation supported our hypothesis that co-culturing of *P. indica* with *P. kessleri*-I induced metabolites which improve the growth of algal cells and neutral lipid biosynthesis inside the cell. However, for further reaffirmation and deep insight into the cellular metabolism, we had performed HPLC and LC–MS–MS based profiling of few essential metabolites [29, 30].

**Table 5:** Metabolites classified in global pathways with their *p* values and FDR values

Pathway name	Total	Hits	*p* value	− log(*p*)	FDR	Impact
Alanine, aspartate and glutamate metabolism	22	3	0.0021	6.1658	0.1827	0.33333
Propanoate metabolism	15	2	0.013831	4.2808	0.38074	0
Nitrogen metabolism	15	2	0.013831	4.2808	0.38074	0
Butanoate metabolism	18	2	0.019721	3.9261	0.38074	0
Selenoamino acid metabolism	19	2	0.021882	3.8221	0.38074	0.00254
Aminoacyl-tRNA biosynthesis	1	3	0.046322	3.0721	0.67166	0
Tropane, piperidine and pyridine alkaloid biosynthesis	8	1	0.095412	2.3495	1	0
Phenylalanine metabolism	8	1	0.095412	2.3495	1	0.5
Sulfur metabolism	12	1	0.13985	1.9672	1	0.13333
Ascorbate and aldarate metabolism	15	1	0.17183	1.7613	1	0.22535
Glyoxylate and dicarboxylate metabolism	17	1	0.19252	1.6475	1	0
Tyrosine metabolism	18	1	0.20269	1.5961	1	0
Citrate cycle (TCA cycle)	20	1	0.22266	1.5021	1	0.04626
Phenylalanine, tyrosine and tryptophan biosynthesis	21	1	0.23247	1.459	1	0
Carbon fixation in photosynthetic organisms	21	1	0.23247	1.459	1	0
Pyruvate metabolism	21	1	0.23247	1.459	1	0.09114
Glycolysis or gluconeogenesis	25	1	0.27056	1.3073	1	0.00147
Glutathione metabolism	26	1	0.2798	1.2737	1	0.07756
Tryptophan metabolism	27	1	0.28893	1.2416	1	0.11765
Porphyrin and chlorophyll metabolism	29	1	0.30686	1.1813	1	0
Arginine and proline metabolism	1	1	0.38246	0.96114	1	0.14004
Phenylpropanoid biosynthesis	1	1	0.43583	0.8305	1	0
Glucosinolate biosynthesis	1	1	0.49812	0.69692	1	0

### Quantification of succinate and hydroxy-glutamate concentration in cell extracts

The concentration of succinate and hydroxy-glutamate (derivatized amino acid) was quantified by High-pressure liquid chromatography (Additional file 1: Figs. S8–S16). The amount of succinate was increased drastically in the co-cultured cells in comparison to control pure algal cultures and fungal cell extracts (Table 6). The enhanced amount of hydroxy-glutamate (Table 7) was also observed in co-cultured cells’ extract, though it was not too high in comparison to succinate accumulation.

**Table 6:** Quantification of succinate concentration in controls and co-cultures samples by HPLC-Aminex

Samples	Succinate amount (µg/mL)
Algal extract	0.39 ± 0.07
Fungal extract	1.00 ± 1.33
Co-cultured cells extract	544.4 ± 45.9

**Table 7:** Quantification of hydroxy-glutamate concentration in controls and co-cultures samples by HPLC-Aminex

Samples	Hydroxy-glutamate amount (µg/mL)
Algal extract	1.6 ± 1.2
Fungal extract	0.11 ± 0.06
Co-cultured cells extract	2.23 ± 1.88

### Multivariate analysis of LC–MS–MS

**Fig. 9:**
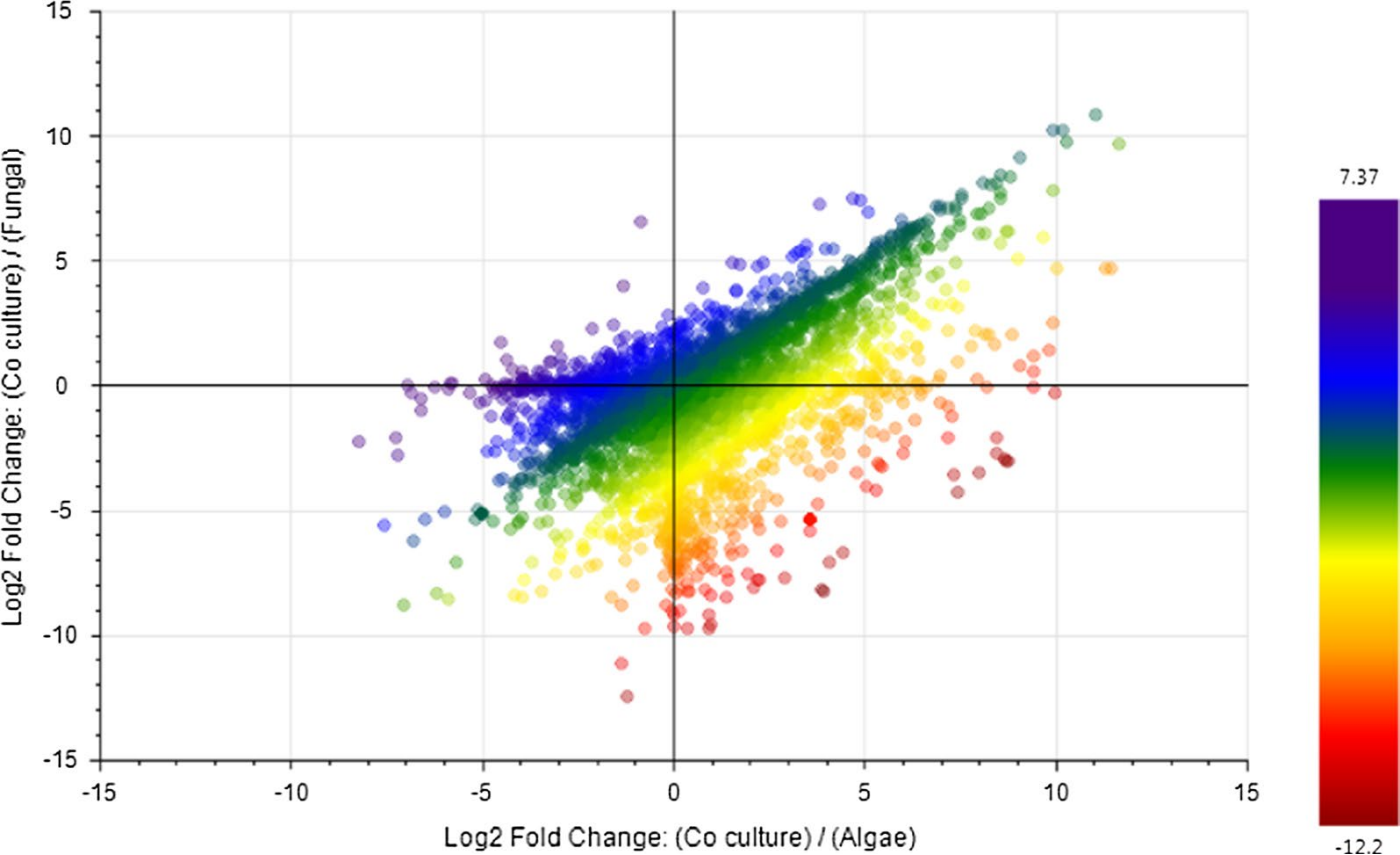
Scatter plot for the compounds exhibiting fold change in the co-culture with respect to individual algal and fungal controls. Each data point in scatter plot represents compound hits and is arranged based on their Log2 fold change for samples in comparison to controls. Changes in the *p* value of compounds hits are shown by color scale. The increase in Log2 fold changes with significant *p* value for compounds mostly belongs to the co-culture samples

We generated the multivariate plots after processing the non-nested LC–MS–MS data by Compound Discoverer 3.0 software. The scatter plot shown in Fig. 9 was generated to depict the fold change difference of each compound hit, while color predicting its *p* value. In this plot, each point represents the compound hit followed by their presence in each data set of sample/control ratio. Based on the scatter plot, we observed that the maximum number of hits belongs to the sample (co-culture) with significantly higher fold change and *p* value having maximum similarity with the compounds found in algal control.

**Fig. 10:**
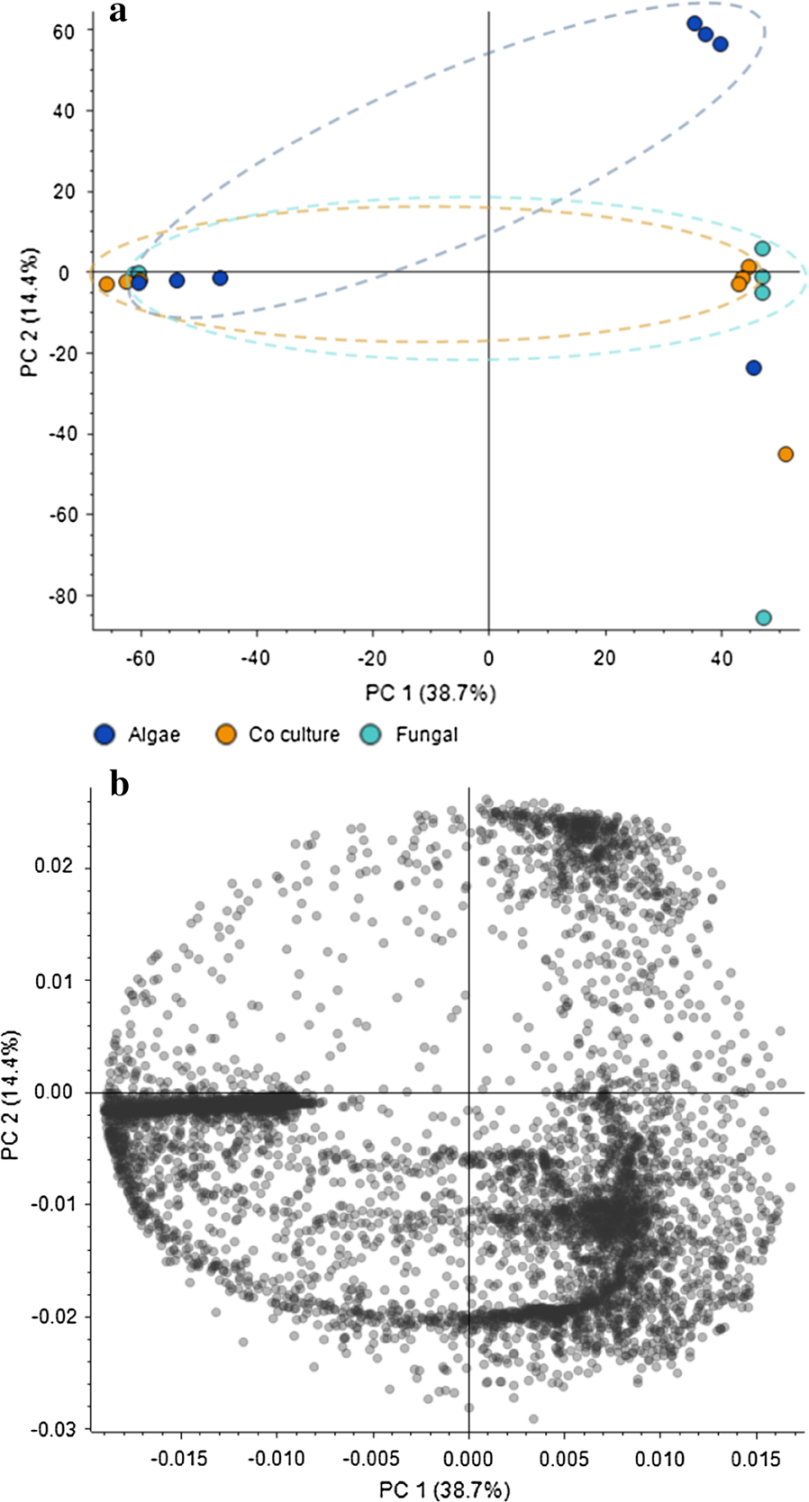
**a**, **b** Principal Component Analysis (PCA) score plot and loading plot; **a** PCA score plot showed the total variance about 53.1% among data sets between each sample group. Dotted line circles are used for grouping the data sets on species profile basis (blue represents algal profile, orange for co-culture, and cyan for fungal profile). Most of the data sets were observed common in each group, only few were diversified. **b** Loading plot represents the region where peaks showed variation with respect to other sample groups and relative to the data set points shown on PCA score plot

**Fig. 11:**
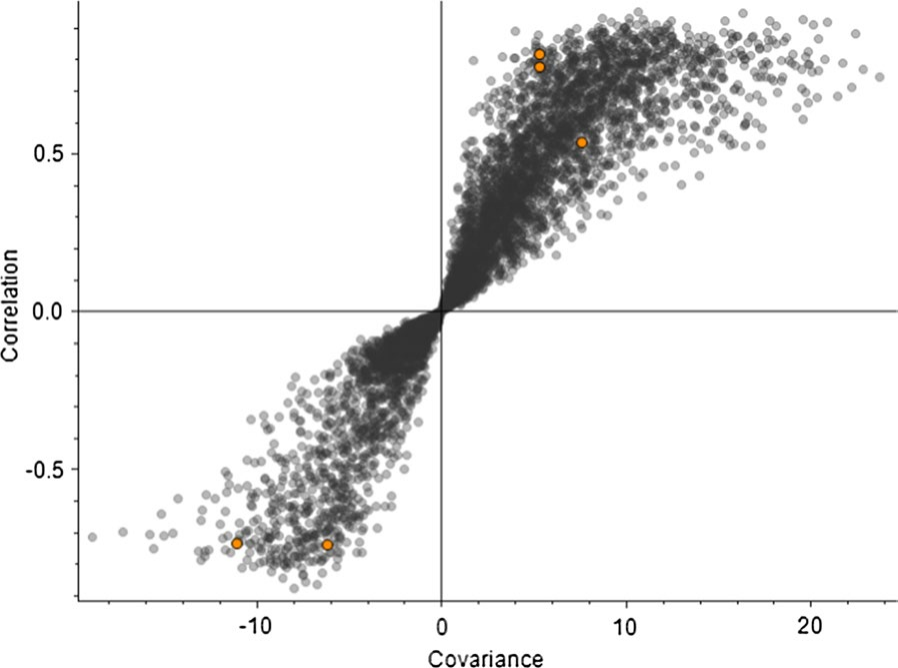
PLSDA (Partial least square discriminant analysis) plot with each data point representing a compound hit. Highlighted data points with orange color are the compounds which were found to be the cause of variation among sample groups as shown in PCA plot

**Fig. 12:**
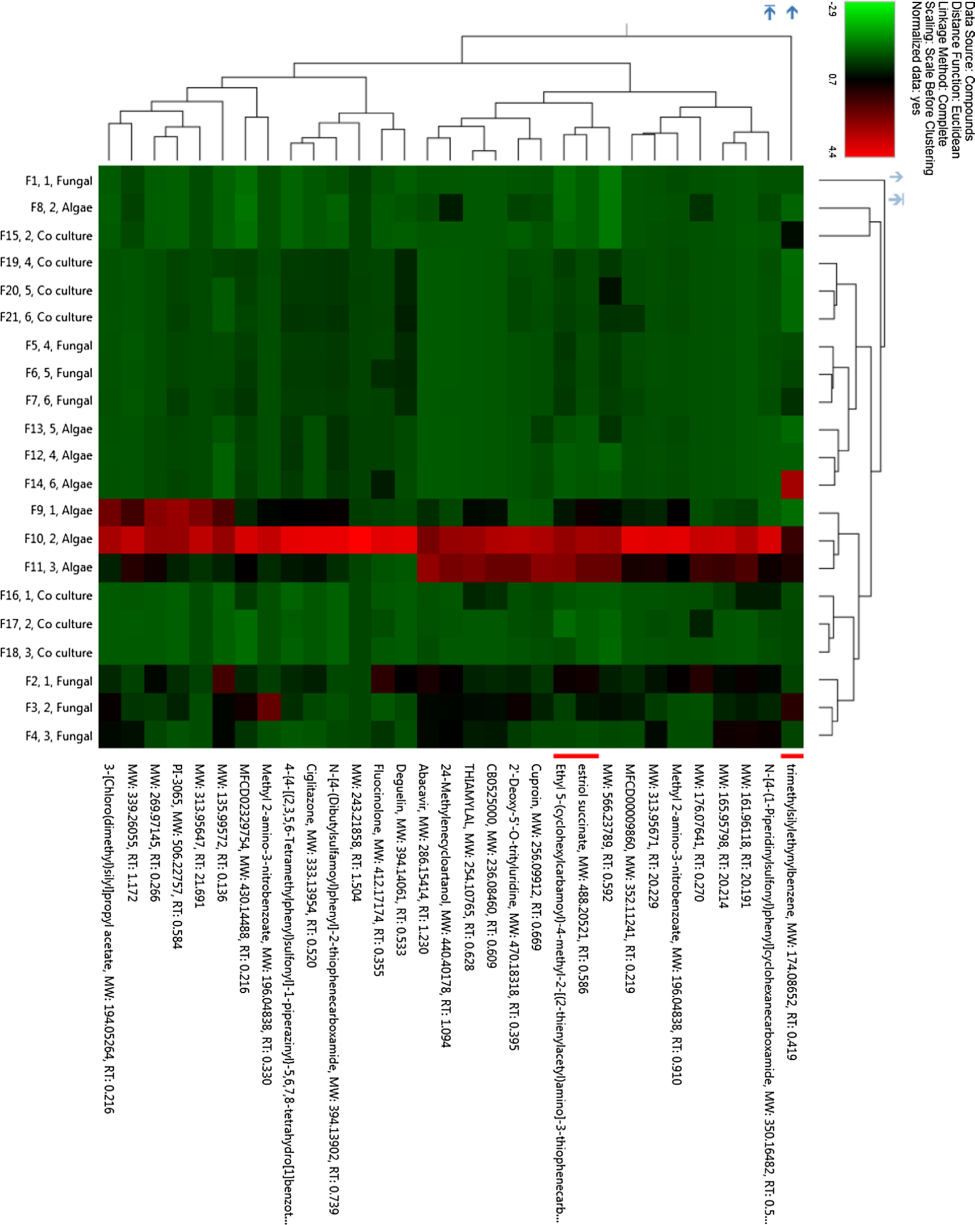
Hierarchical cluster analysis visualization through heat map showing significantly high *p* value for succinate compound in co-culture extracts (highlighted with red color)

**Fig. 13:**
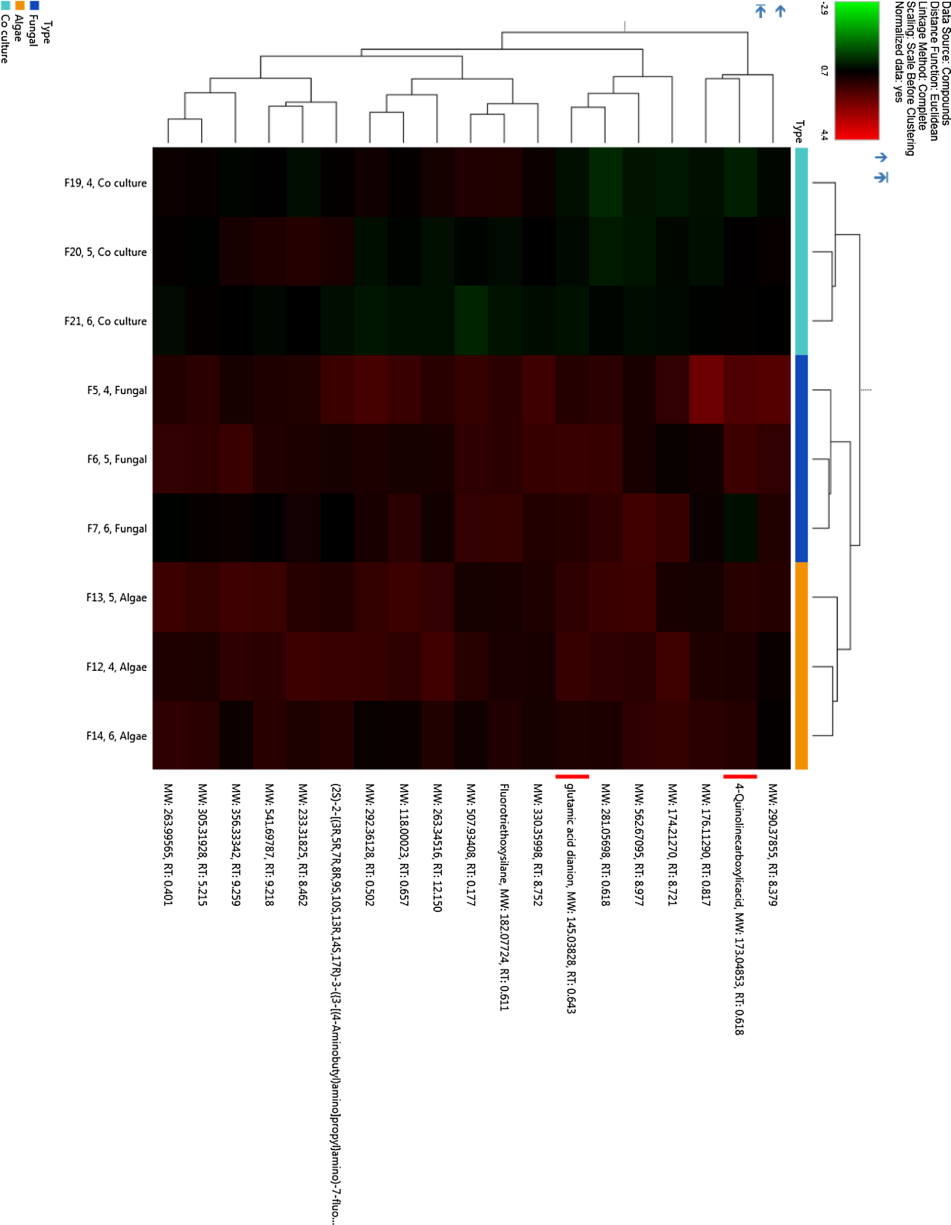
Hierarchical cluster analysis visualization through heat map showing significantly high *p* value for glutamate and carboxylic acid in co-culture extracts (highlighted with red color)

**Fig. 14:**
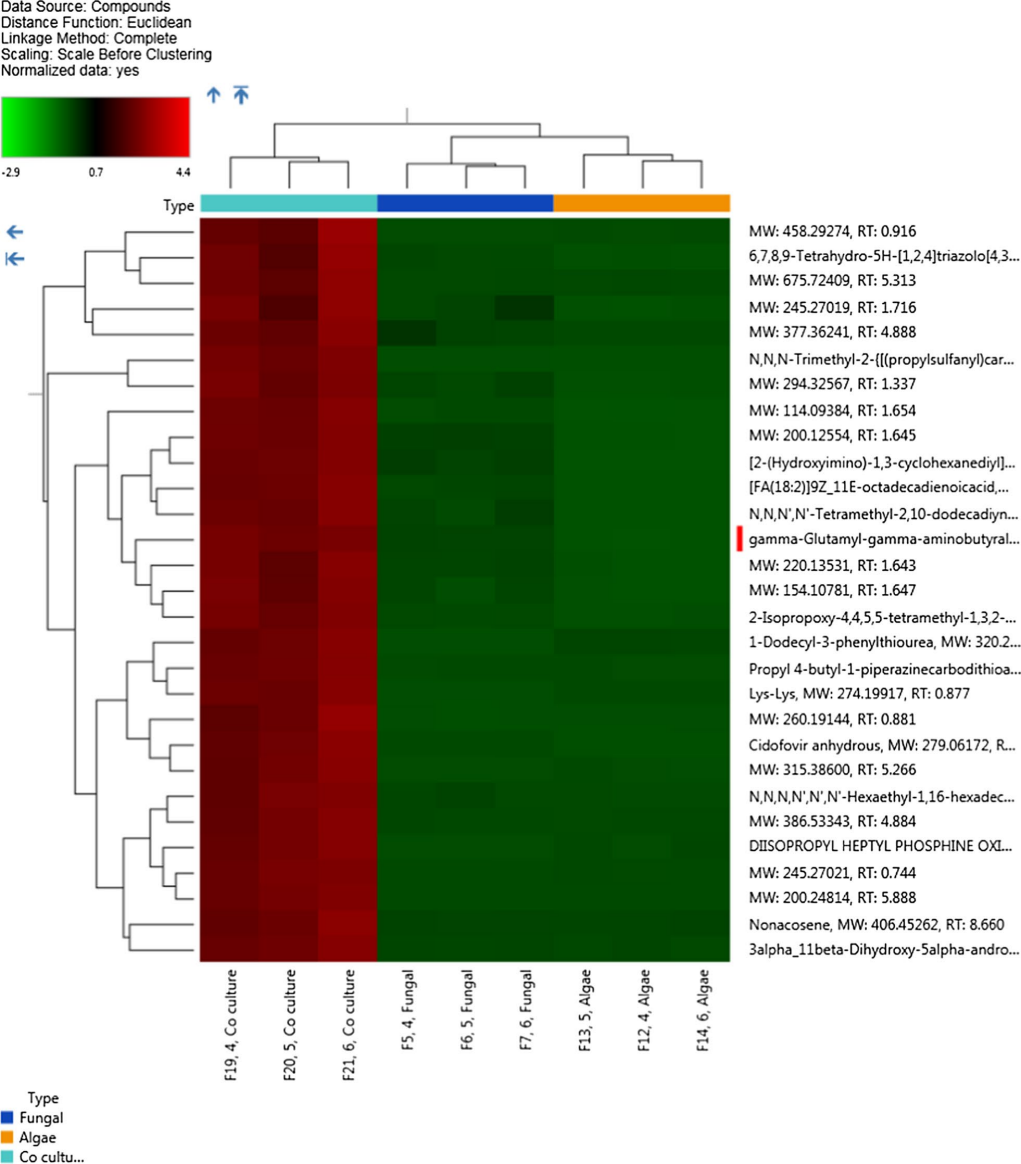
Hierarchical cluster analysis visualization through heat map showing significantly high *p* value for GABA (Gamma Amino glutamyl butyrate) in co-culture extracts (highlighted with red color)

**Fig. 15:**
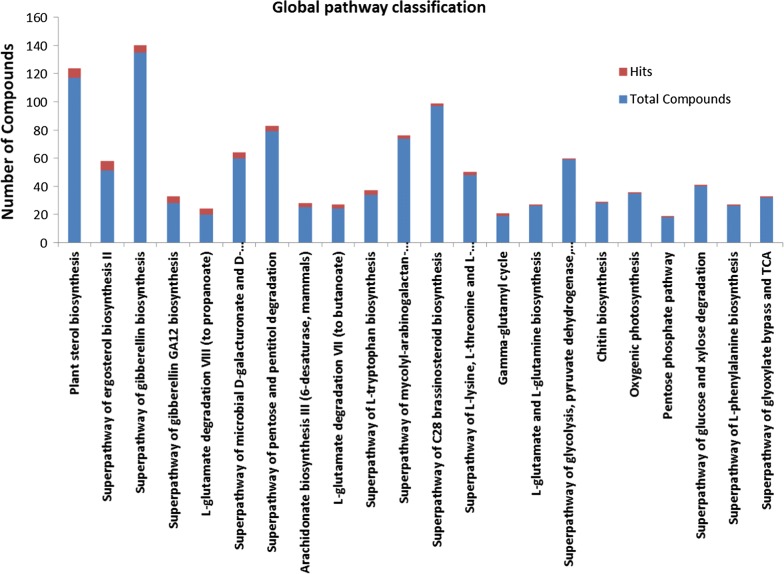
Global metabolic pathway classification of LC–MS–MS metabolomics data of *P. indica*–*P. kessleri*-I co-culture. Total number of bioactive metabolites identified in co-culture of *Pi* and *Pk*-I (*P. indica* and *P. kessleri*-I), positioned in global metabolic pathways using Metabolika database of Discoverer 3.0

Scoring plot generated simultaneously with loading plot during Principal Component Analysis (PCA) was used to group the compound hits together based on sample/control profiles. The PCA score plot also depicted the presence of three separate data set points (containing alga, fungus and co-culture shown in Fig. 10a, b). The PCA score plot, generated to categorize between three profiles, has showed total variance of 53% among the compound data set points. In our multivariate study, the higher fold change was observed in the compound hits with a significant *p* value particularly in case of co-culture. Further, Partial Least Squares Discriminant Analysis (PLS-DA) plot was generated to differentiate the varying compound hits of groups’ profile. In PLS-DA plot, varying compound hits of groups were highlighted with orange color (Fig. 11). The above data set points were also visualized through the hierarchical clustered analysis of a heatmap. We have observed a significant increase in the *p* value of succinate (Fig. 12), glutamate (Fig. 13), and GABA (gamma-aminobutyric acid) (Fig. 14). Bioactive compound hits were detected using LC–MS–MS study to support the GC–MS data shown in Additional file 1: Figs. S17. Detected compound hits in LC–MS–MS metabolomic analysis were classified into their global pathways by Metabolika database linked in Compound Discoverer 3.0 software (Fig. 15). We observed that maximum number of compound hits belonged to carbon metabolism and amino acid biosynthesis pathways. Thus, using LC–MS–MS study, the role of succinate and glutamate was reaffirmed in enhancing the metabolic pathways’ efficiency related to growth modulation in *P. kessleri*-I when co-cultured with *P. indica*.

## Discussion

Owing to interaction with the endophytic fungal strain, the microalga adapts to the biotic stress and usually undergoes a series of changes at the metabolic levels. Indeed, the linkage between the two species has been also confirmed by the double-layer semi-solid agar culturing, where the agar plates that co-cultured *P. kessleri*-I showed higher number of colonies with respect to control plates (Additional file 1: Fig. S18 A, B). The associated metabolic changes cause the modulation in the algal growth and lipid profile. Henceforth, deciphering the metabolic profile under stress conditions will be crucial for acquiring the interaction knowledge of the unicellular photoautotrophs with fungal species. Association of endophytic fungal strains with plants or other species has gained attention due to its synergistic and commensal effects. Such studies give insight toward the dynamic relation of cell–cell interaction and its effect at physiological scale [37, 38]. Thus, in our study, we have tried to elucidate the effect of *P. indica* on *P. kessleri*-I biomass, lipid profile and associated metabolic changes. Hua and co-authors previously reported the effect of the colonization of Chinese cabbage by *P. indica*, and revealed the increase in metabolites associated with the TCA (tri-carboxylic acid) cycle of the host organism [39]. There was also an increase in the intermediate metabolites related to GABA (gamma-aminobutyric acid) synthesis, Tryptophan, and Phenylalanine metabolism. On a similar note, we have also observed an increase in the abundance percentage of TCA cycle-related metabolites like succinate and acetate. The reaction of acetate with coenzyme A (CoA), catalyzed by enzyme Acetyl CoA synthetase, helps in the formation of Acetyl CoA which is involved in many biochemical reactions, especially energy production [40]. Succinate is a central metabolite since it is an intermediate of TCA cycle and GABA shunt as well as linked with amino acid biosynthesis pathway via the formation of pyruvate or oxaloacetate [41].

Further, it has been observed that both glutamate and succinate levels are increased, which are major components of the GABA shunt. Therefore, they play major roles in nitrogen metabolism and primary carbon metabolism [42, 43]. There are three catalytic conversion steps in GABA shunt which are as follows: (i) glutamate decarboxylase enzyme catalytically converts l-glutamate into ɤ-aminobutyric acid (GABA) (ii) GABA is converted to succinate semialdehyde by the action of GABA transaminase (iii) finally, succinic semialdehyde dehydrogenase enzyme converts succinate semialdehyde to succinate [43, 44]. Increase in the abundance of glutamate could be the probable result of the diversion of the TCA cycle to glutamate formation through citrate thus leading to a decrease in the abundance 2-hydroxy butane. Several studies based on plants have evidenced an increase in growth due to GABA shunt internetworking with carbon/nitrogen balance [45]. Increase in the GABA level is an outcome of biotic or abiotic stress on plant tissues. These outcomes from metabolic profiling depict that modulation of biomass and lipid is the effect of induction of intermediate metabolites of GABA shunt and carbon metabolism that play major roles in sequestering carbon, nitrogen and phosphate inside an algal cell. In our LC–MS–MS-based metabolomics study, we have observed maximum hits related to compounds having phosphate as their functional group. We also observed the abundance of l-glutamate and succinate in the intracellular extract of co-culture cells in comparison to controls. Other then deciphering the changes occurring at metabolic, physical and morphologically level, the harvesting efficiency of mixed *P. indica* pellets with *Parachlorella kessleri*-I at pH 3 after an incubation of ~ 72 h evidenced for ~ 60% harvesting efficiency (Additional file 1: Fig. S19). However, further detailed analysis at the proteome and transcriptome level needs to be performed in order to elucidate the comprehensive molecular changes of algae under the commensal influence of an endophytic fungus.

## Concluding remarks

Based on the study carried out to observe the effect of *P. indica* on *P. kessleri*-I, we concluded the existence of commensalism between them that helps *P. kessleri*-I in the modulation of biomass and FAME content. This modulation is an effect of directed changes or enhancement in intracellular metabolic networks. We observed the changes in the central backbone of the carbon metabolic pathway comprising the inter-networks of carbon fixation, pyruvate metabolism, TCA pathway and stress-based propionate metabolism pathway. Thus, this study provided the strong evidence that interaction between the fungal and the algal cells modulated the growth and lipid accumulation of the latter. Thus, the endophytic fungus co-culturing technique may serve as an important advancement for biofuel industries to enhance the biomass, and thus the lipid content of algal strains at commercial level.

## Additional file

**Additional file 1.** LCMS and GCMS parameters + Multivariate analysis of GCMS.

## Data Availability

All data generated or analyzed during this study are included in this published article and its additional files.
