# Supplementary material for: Amelioration of biomass and lipid in marine alga by an endophytic fungus Piriformospora indica
Source: Biotechnol Biofuels. 2019 Jul 8;12:176. doi: 10.1186/s13068-019-1516-6 (PMC6613240; doi:10.1186/s13068-019-1516-6)
Supplement: Supplementary file 1 — Additional file 1. LCMS and GCMS parameters + Multivariate analysis of GCMS. [file 13068_2019_1516_MOESM1_ESM.doc]

**Additional file 1**

**Amelioration of biomass and lipid in marine alga by an endophytic fungus *Piriformospora indica***

**Vipul Swarup Bhatnagar^1^, Prasun Bandyopadhyay^1^, Girish H. Rajacharya^1^, Sharanya Sarkar, Krishna Mohan Poluri^2^, Shashi Kumar^1^**

**^1^International Centre for Genetic Engineering and Biotechnology, Aruna Asaf Ali Marg, New Delhi 110067**

**^2^Department of Biotechnology & Centre for Transportation Systems (CTRANS), Indian Institute of Technology Roorkee, Roorkee - 247667 Uttarakhand, India**

**Correspondence to:** [**skrhode@icgeb.res.in**](mailto:skrhode@icgeb.res.in)

**Table S1:** Methods and parameters for Orbitrap Fusion Lumos (LC-MS-MS).

| Cuztomised calibration ions* | |
| --- | --- |
| Positive | Negative |
| 74.09643 | 68.99576 |
| 110.07127 | 96.9601 |
| 138.06619 | 112.98453 |
| 195.08765 | 265.1479 |
| 262.63612 | 514.2844 |
| 524.26496 | 1180.0036 |
| 1121.99702 |  |
| 1221.99064 |  |

| HESI parameters | | |
| --- | --- | --- |
| Ion source type: H-ESI | Positive | Negative |
| Voltage (V) | 3500 | 2500 |
| Sheath gas (arb) | 42 | 42 |
| Aux gas flow (arb) | 10 | 10 |
| Sweep gas flow (arb) | 1 | 1 |
| S-lens RF | 40 | 40 |
| Capillary temp (⁰C) | 340 | 340 |
| Aux gas heater temp (⁰C) | 360 | 360 |

| MS-MS Method | | |
| --- | --- | --- |
| Scan-mode | Resolution | Injection Time (ms) |
| Fullscan | 60,000 | 50 |
| ddMS2 (Orbitrap) | 15,000 | 22 |

| Mobile phase | | |
| --- | --- | --- |
|  | A | B |
| Mix mode (Acclaim Trinity P2) | 10mM AcONH4 in H_2_O (pH 5.0) | ACN : 100mM AcONH4 in H_2_O (60% /40%) pH 5.0 |

|  | **Retention (min)** | **Flow (mL/min)** | **%B** |
| --- | --- | --- | --- |
| **1** | -5.0 | 0.35 | 0.0 |
| **2** | 0.0 | 0.35 | 0.0 |
| **3** | 20.0 | 0.35 | 100.0 |
| **4** | 22.0 | 0.35 | 100.0 |

**Table S2:** UATR wavenumber hits for Algae (Control)

| **Wavenumber (cm^-1^)** | **Peak Intensity** | **Group** |
| --- | --- | --- |
| 3297.64 | 0.2499 | Amine A&B; N-H stretch |
| 1637.96 | 0.1314 | Amide 1 (β-sheet); C=O stretch |

**Table S3:** UATR wavenumber hits for Fungus (Control)

| **Wavenumber (cm^-1^)** | **Peak Intensity** | **Group** |
| --- | --- | --- |
| 3236.81 | -0.7713 | Amine A&B; N-H stretch |
| 1630.59 | -0.2941 | C=O; stretch Amide 1 |
| 1033.01 | -0.1639 | C-O-C vibration of Polysaccharide |

**Table S4:** UATR wavenumber hits for Algae + Fungus Co-pelletization

| **Wavenumber (cm^-1^)** | **Peak Intensity** | **Group** |
| --- | --- | --- |
| 1637.55 | 0.1495 | Amide 1 (β-sheet); C-O stretch |
| 587.21 | 0.4646 | C=O bending |
| 575.34 | 0.4827 | C-O bending |

Presence of C-O stretching and bending bonds of carbohydrate vibration with slightly higher peak intensity in case of co-culture than in pure cultures of *P. kessleri*-I and *P. indica* indicates the presence of cell-cell interaction which provides us with the evidence that there is commensal relation between endophytic fungus *P. indica* and marine algae *P. kessleri*-I*.* Also, all the samples have peaks in the spectral region of 1500-1800 cm^-1^ due to the absorbance by peptide backbone. The peak intensity of amide bond formed in case of *P. kessleri*-I (control) is more in comparison to the peak intensity of amide functional group detected in case of alga-fungus conjugated pellets. The intensity in case of the co-culture is quite near to the amide peak intensity detected in case of fungus pellet extract. This can be due to the nutrition exchange and utilization of peptide by fungus from algal cell.

**Table S5:** Induced and Down regulated bioactive metabolites in co-cultured *P. kessleri*-I and *P. indica* metabolome profile with respect to abundance value of controls.

| **S.No.** | **Metabolite** | **A** | **F** | **AF** | **Fold change = AF - (A+F)** |
| --- | --- | --- | --- | --- | --- |
| 1 | Glutamic acid | ND | ND | 0.89% + 0.05% | 0.89% |
| 2 | Butanedioic acid | ND | 0.43% + 0.03% | 0.82% + 0.04% | 0.40% |
| 3 | Propanoic acid | ND | ND | 0.36% + 0.19% | 0.36% |
| 4 | quinazoline | ND | ND | 0.41% + 0.25% | 0.41% |
| 5 | phosphate | ND | ND | 0.54% + 0.21% | 0.54% |
| 6 | l-Alanine | 0.20% + 0.02% | ND | 0.65% + 0.27% | 0.44% |
| 7 | Acetic acid | ND | ND | 0.77% + 0.17% | 0.77% |
| 8 | Propane | 0.44% | 0.59% + 0.24% | 0.42% + 0.16% | -0.62% |
| 9 | Benzaldehyde | 0.19% + 0.07% | 0.26% + 0.22% | 0.16% + 0.32% | -0.29% |
| 10 | Acetamide | 0.40% + 0.21% | 0.24% + 0.05% | 0.73% + 0.14% | 0.09% |
| 11 | amine | ND | 0.41% + 0.21% | 0.39% + 0.22% | -0.02% |
| 12 | Isoindole | 0.22% + 0.03% | 0.30% + 0.07% | 0.18% + 0.02% | -0.33% |
| 13 | Galactopyranose | 0.12% + 0.02% | 0.36% + 0.21% | 0.16% + 0.10% | -0.32% |
| 14 | Glucopyranose | 0.15% + 0.04% | 0.41% + 0.16% | 0.18% + 0.10% | -0.38% |
| 15 | Indole-3-acetic acid | 0.20% + 0.13% | ND | 0.19% + 0.12% | -0.01% |
| 16 | 1,3-propanediol | 0.63% + 0.36% | 0.16% + 0.02% | 0.11% + 0.01% | -0.68% |
| 17 | Thiazolidine-4-carboxylic acid | 0.22% + 0.03% | ND | 0.16% + 0.02% | -0.06% |
| 18 | Benzoic acid | 0.16% + 0.04% | 0.29% + 0.20% | 0.52% + 0.28% | 0.08% |
| 19 | acetone | 0.23% + 0.15% | 0.19% + 0.05% | ND | -0.42% |

**Induced and Down regulated bioactive metabolites:** Abundance relative percentage for a metabolite in each library is calculated by taking the average of abundance % of particular metabolites of all the detected hits among each of the 18 profiles in 1 library. Fold change for a metabolite in co-culture is determined by subtracting its average from the sum of its average calculated in controls.

Abundance fold change = Abundance of metabolite in AF [algae + fungi co-culture] – (Abundance of metabolite in alga + fungal hyphae)

**Fig. S1:** FTIR-UATR transmission spectra for Algae (*P. kessleri*-I) Control

**Fig. S2:** FTIR-UATR transmission spectra for Fungus (*P. indica*) Control

**Fig. S3** FTIR-UATR transmission spectra for Algae + Fungus Co-pelletization

**Fig. S4:** Standard curve between dry cell weight and its total chlorophyll content for pure *P. kessleri*-I culture where red line depicts the validation of co-culture experimental data


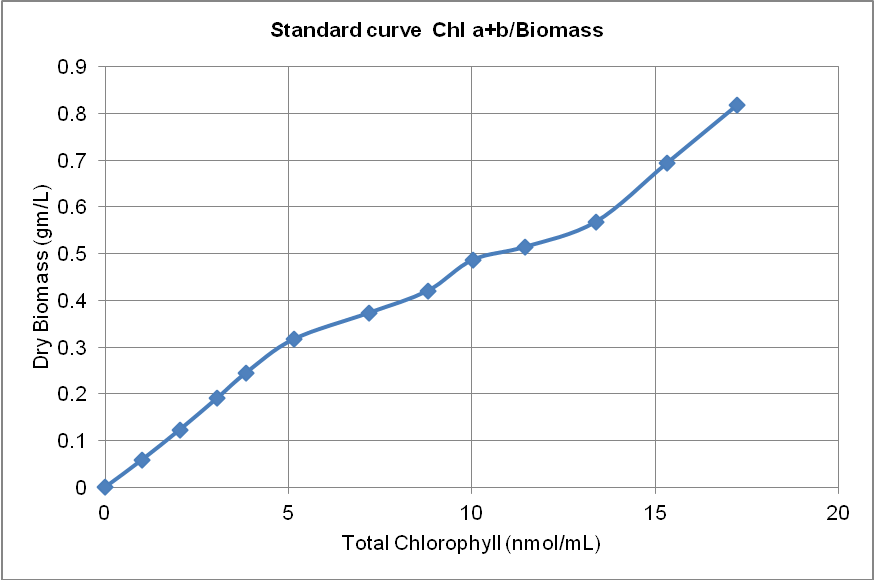


**Fig. S5:** Scores plot of principal components analysis shows the correlation between sample types. Each green dot represents co-culture metabolites, blue colour represents algal metabolites and fungal metabolites are denoted by black dots.


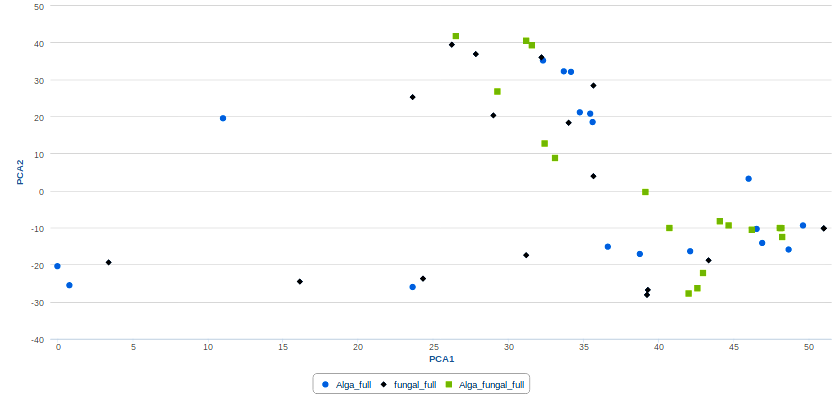


**Fig. S6:** Interactive Principal Component Analysis Loading plot shows the relation between the sample types and the relationship between metabolites. It is observed that there is 60% variance among the sample metabolites in 2D-PCA on the basis of spectral and feature intensity of metabolites.


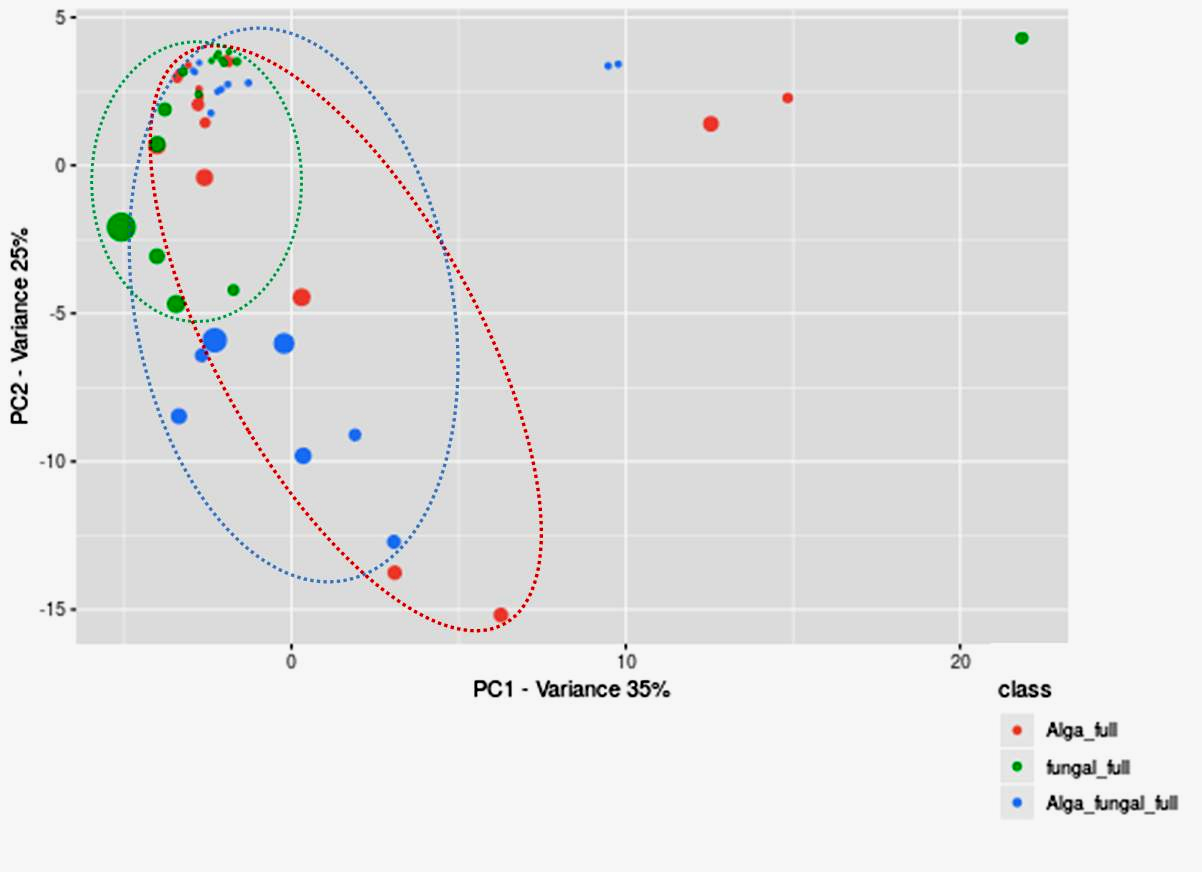


**Fig. S7**: HPLC Chromatogram of blank on Aminex column.


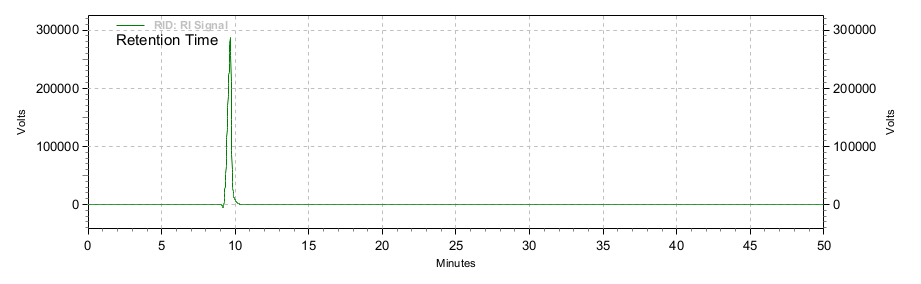


**Fig. S8**: HPLC Chromatogram of succinate standard (1mg/mL) at RT of 24.394 minutes on Aminex column.


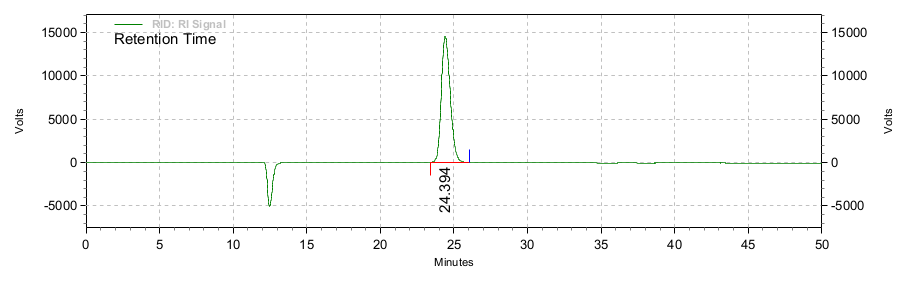


**Fig. S9**: HPLC Chromatogram of pure algal cells extract showing succinate peak at RT 24.498 on Aminex column.


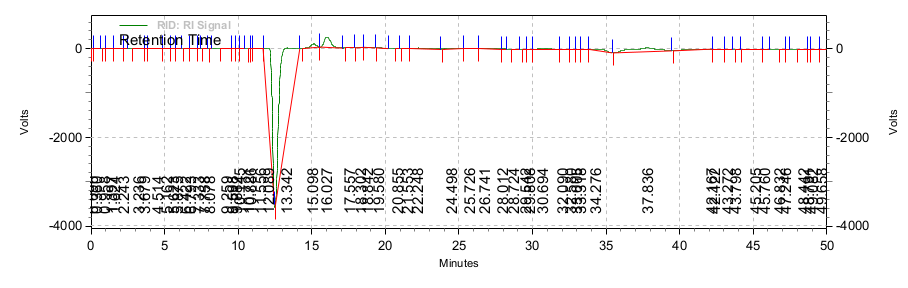


**Fig. S10**: HPLC Chromatogram of pure Fungal hyphae extract showing succinate peak at RT 24.660 on Aminex column.


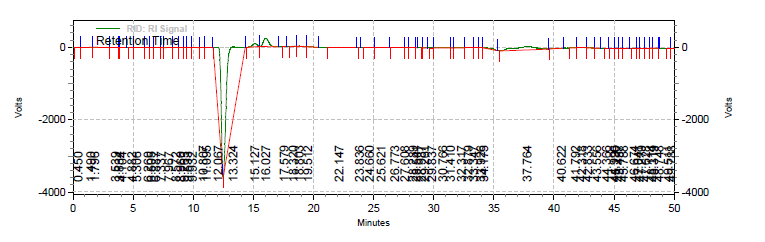


**Fig. S11**: HPLC Chromatogram of pure co-cultured cells extract showing succinate peak at RT 24.379 on Aminex column.


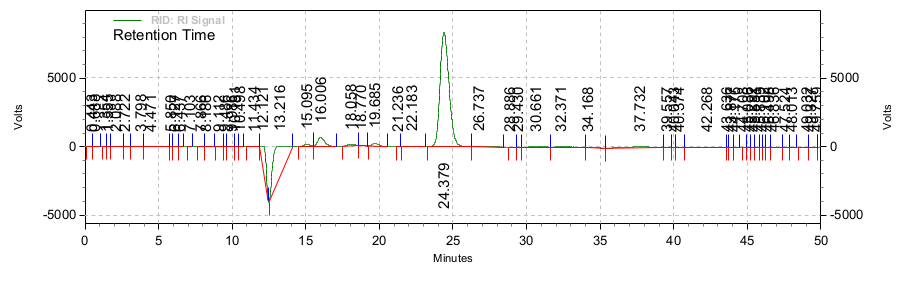


**Fig. S12**: HPLC Chromatogram of hydroxy glutamate standard (1mg/mL) at RT of 28.375 minutes on Aminex column.


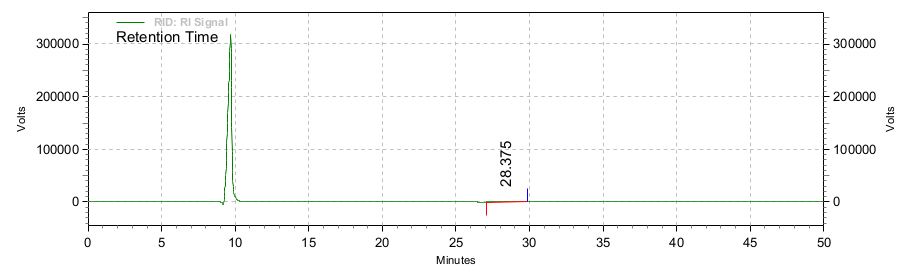


**Fig. S13**: HPLC Chromatogram of pure algal cells extract showing hydroxy glutamate peak at RT 28.465 on Aminex column.


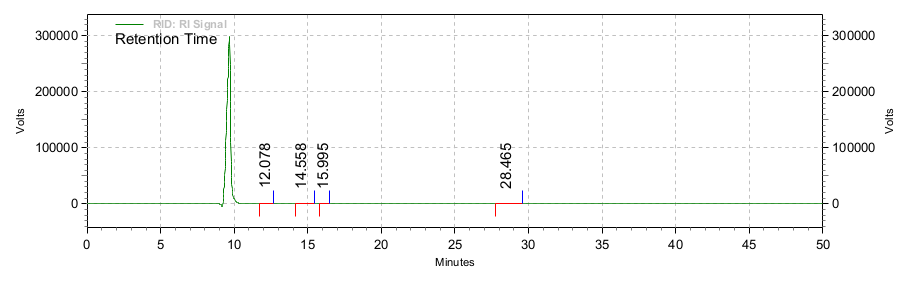


**Fig. S14**: HPLC Chromatogram of pure fungal hyphae extract showing hydroxy glutamate peak at RT 28.422 on Aminex column.


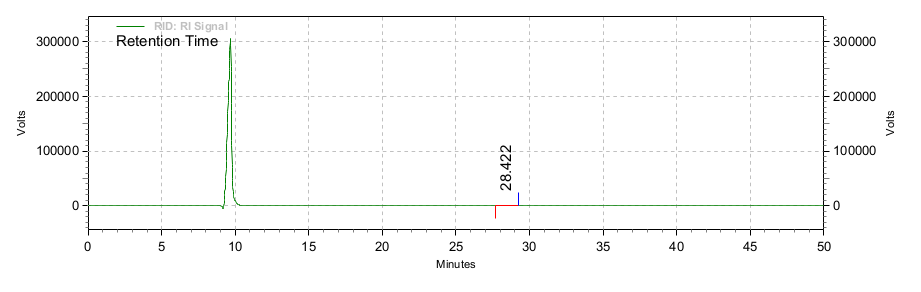


**Fig. S15:** HPLC Chromatogram of co-cultured cells extract showing hydroxy glutamate peak at RT 28.498 on Aminex column.


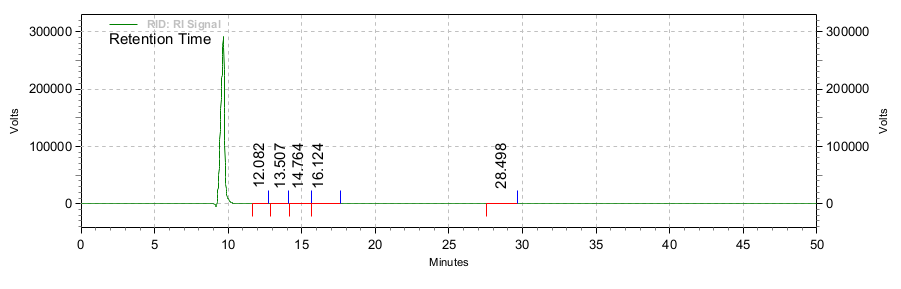


**Fig. S16:** Chromatogram of Internal Standard Heptadecanoic methyl ester used in GC-MS.

**Fig. S17:** Bioactive metabolites depicted from LC-MS based metabolomic analysis observed to be common with GC-MS metabolomic analysis depicted with their p-values and fold change.


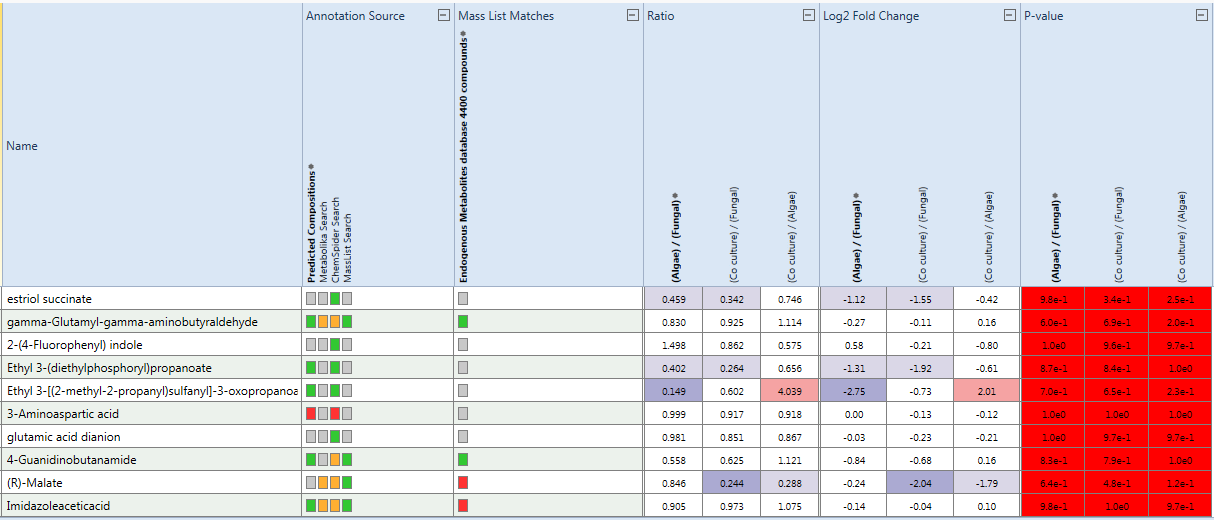


**Fig. S18:** Estimating harvesting efficiency of *Piriformospora indica* and pelleted algal cell culture with fungi. The *P. indica* paraglobules were mixed with the alga *Parachlorella kessleri*-I using acidic and alkaline pH 3 and 7 respectively for 72hrs approx. The *P. indica* paraglobules were observed efficiently trapping the *P. kessleri*-I at the acidic pH 3. Control without any para-globules. (B) Harvested algal cells using *P. indica* paraglobules


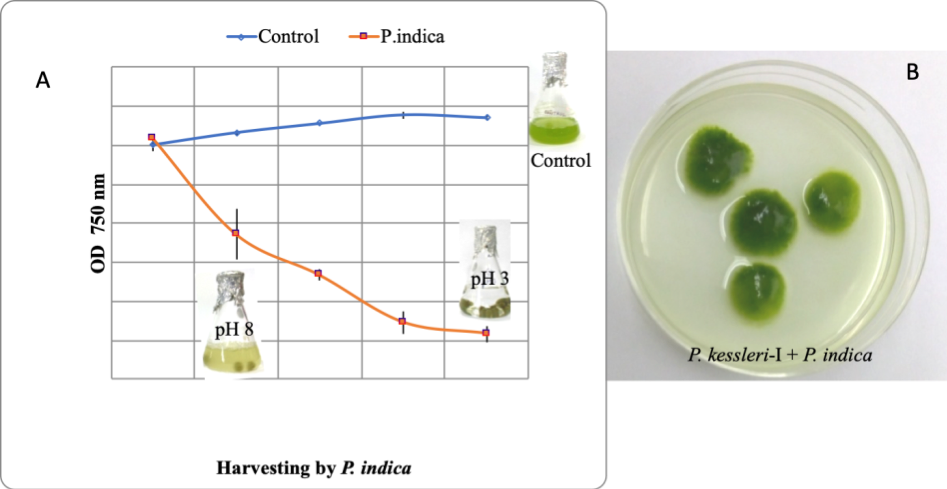


**Fig. S19 A-B:** Double layer agar spread plating of *P. kessleri*-I (control) with *P. indica* to observe the growth difference and mutual relationship between two species. **(A)** *P. kessleri*-I was plated as pure culture (as control) on semi solid TAP-agar plate and incubated for 7 days. **(B)**  The 7 days grown *P. indica* co-cultured with *P. kessleri*-I on semi solid agar medium, incubated for another 7 days.


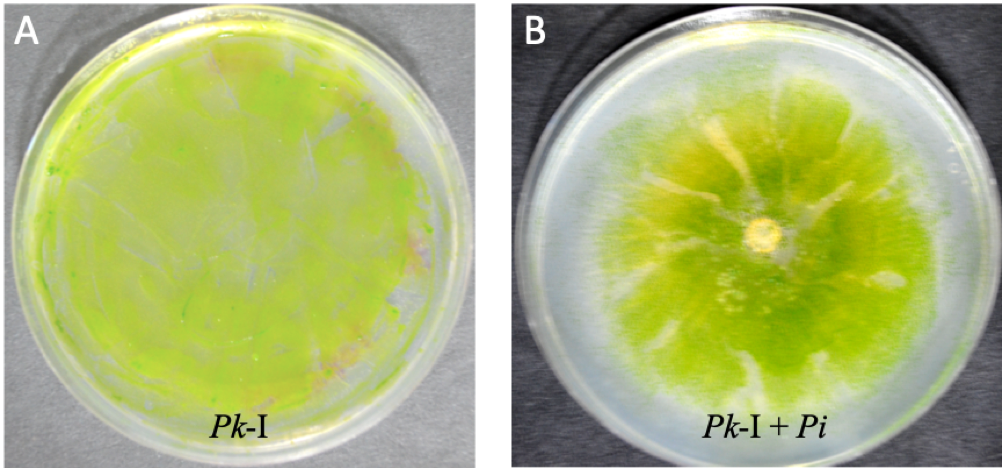


**Methods and Parameters for GC-MS**

**FAME analysis by Omega wax:**

A Gas Chromatography Mass spectrometry (GC MS) analysis of Fatty acid methyl esters was performed using Agilent 7890A series GC system equipped with a Omegawax column (30m*0.25mm ID, 0.25um thickness, SUPELCO) coupled with a Agilent 7000 QQQ MS . Omegawax column was used for good separation and for gas chromatography mass spectroscopic detection. Electron ionization system with ionization energy of 70eV was used, 99.99 % pure helium gas was used as a carrier gas at a constant flow rate of 1.3 ml/min. Mass transfer line and injector temperature were set at 220˚C and 250˚C respectively, and the oven temperature was programmed, initial temperature was 40 °C for 2 min then 10 °C/min to 280 °C for 2 min. 2 μl of sample was injected in the splitless mode. The signals were recorded in full scan mode (m/z 20-600, 200 scan/milli seconds). All components were identified by comparing their mass spectra with those obtained from authentic samples and/or the NIST05 mass spectral database.

**Untargeted Metabolomics by HP-5**

A Gas Chromatography Mass spectrometry (GC MS) analysis of untargeted metabolomic samples were performed using Agilent 7890A series GC system equipped with a HP-5 column (30m*0.25mm ID, 0.25um thickness, Varian) coupled with a Agilent 7000 QQQ MS . HP-5 column was used for good separation and for gas chromatography mass spectroscopic detection. Electron ionization system with ionization energy of 70eV was used, 99.99 % pure helium gas was used as a carrier gas at a constant flow rate of 1.1 ml/min. Mass transfer line and injector temperature were set at 220˚C and 250˚C respectively, and the oven temperature was programmed, initial temperature was 60 °C for 1 min then 5 °C/min to 180 °C for 1 min then 10 °C/min to 310 °C for 2 min. 1 μl of sample was injected in the split mode 5:1. The signals were recorded in full scan mode (m/z 20-600, 250 scan/milli seconds). All components were identified by comparison of their mass spectra with those obtained from authentic samples and/or the NIST mass spectral database using AMDIS and mass hunter software.

**HPLC Specification's for Succinate Estimation**

**Instrument**: Agilent 1260 Series (Binary Pump System)

**Method Parameters:**

**Pump**: Binary Pump

**Mobile Phase**: 4mM H_2_SO_4_

**Flow rate** : 0.3 ml/min

**Run Time:** 50 min

**Succinate Elution Time**: ~ 24.39 min

**Column:** Aminex HPX 87 H (300*7.8mm, 10u)

Ion Exchange column

Bio-Rad (Cat. No: 125-0140), With respective Guard Column

Column Temperature: 40 ºC

**Detector:** Refractive Index Detector (RID)

Attenuation: 500000 nRIU

Signal Polarity: Positive

**HPLC Specification's for hydroxyl-glutamate Estimation**

**Instrument**: Agilent 1260 Series (Binary Pump System)

**Method Parameters:**

**Pump**: Binary Pump

**Mobile Phase**: 4mM H_2_SO_4_

**Flow rate** : 0.4 ml/min

**Run Time:** 50 min

**hydroxyl-glutamate Elution Time**: ~ 28.375 min

**Column:** Aminex HPX 87 H (300*7.8mm, 10u)

Ion Exchange column

Bio-Rad (Cat. No: 125-0140), With respective Guard Column

Column Temperature: 40 ºC

**Detector:** Refractive Index Detector (RID)

Attenuation: 500000 nRIU

Signal Polarity: Positive

**Compound Discoverer (3.0) Methods and parameters**

**Work Flow:**

Search name: non-nested metab

Search description: Untargeted Metabolomics workflow: Find and identify the differences between samples.

- Performs retention time alignment, unknown compound detection, and compound grouping across all samples. Predicts elemental compositions for all compounds, fills gaps across all samples, and hides chemical background (using Blank samples). Identifies compounds using mzCloud (ddMS2) and ChemSpider (formula or exact mass). Also performs similarity search for all compounds with ddMS2 data using mzCloud. Maps compounds to biological pathways using Metabolika. Applies QC-based batch normalization if QC samples are available. Calculates differential analysis (t-test or ANOVA), determines p-values, adjusted p-values, ratios, fold change, CV, etc.).

Created with Discoverer version: 3.0.0.294

[Input Files (6)]

-->Select Spectra (33)

[Select Spectra (33)]

-->Align Retention Times (26)

[Align Retention Times (26)]

-->Detect Compounds (9)

[Detect Compounds (9)]

-->Group Compounds (31)

[Group Compounds (31)]

-->Assign Compound Annotations (25)

-->Fill Gaps (32)

-->Predict Compositions (29)

-->Search ChemSpider (23)

-->Map to Metabolika Pathways (34)

-->Search Mass Lists (35)

[Fill Gaps (32)]

-->Normalize Areas (30)

[Normalize Areas (30)]

Processing node 33: Select Spectra

------------------------------------------------------------------

1. General Settings:

- Precursor Selection: Use MS(n - 1) Precursor

- Use Isotope Pattern in Precursor Reevaluation: True

- Provide Profile Spectra: Automatic

- Store Chromatograms: False

2. Spectrum Properties Filter:

- Lower RT Limit: 0

- Upper RT Limit: 0

- First Scan: 0

- Last Scan: 0

- Ignore Specified Scans: (not specified)

- Lowest Charge State: 0

- Highest Charge State: 0

- Min. Precursor Mass: 0 Da

- Max. Precursor Mass: 5000 Da

- Total Intensity Threshold: 0

- Minimum Peak Count: 1

3. Scan Event Filters:

- Mass Analyzer: (not specified)

- MS Order: Any

- Activation Type: (not specified)

- Min. Collision Energy: 0

- Max. Collision Energy: 1000

- Scan Type: Any

- Polarity Mode: (not specified)

4. Peak Filters:

- S/N Threshold (FT-only): 1.5

------------------------------------------------------------------

Processing node 26: Align Retention Times

------------------------------------------------------------------

1. General Settings:

- Alignment Model: Adaptive curve

- Alignment Fallback: Use Linear Model

- Maximum Shift [min]: 2

- Shift Reference File: True

- Mass Tolerance: 5 ppm

- Remove Outlier: True

------------------------------------------------------------------

Processing node 9: Detect Compounds

------------------------------------------------------------------

1. General Settings:

- Mass Tolerance [ppm]: 5 ppm

- Intensity Tolerance [%]: 30

- S/N Threshold: 3

- Min. Peak Intensity: 100000

[M+H-H2O]+1

- Base Ions: [M+H]+1; [M-H]-1

- Min. Element Counts: C H

2. Peak Detection:

- Filter Peaks: True

- Max. Peak Width [min]: 0.5

- Remove Singlets: True

- Min. # Scans per Peak: 5

- Min. # Isotopes: 1

------------------------------------------------------------------

Processing node 31: Group Compounds

------------------------------------------------------------------

1. Compound Consolidation:

- Mass Tolerance: 5 ppm

- RT Tolerance [min]: 0.2

2. Fragment Data Selection:

- Preferred Ions: [M+H]+1; [M-H]-1

------------------------------------------------------------------

Processing node 25: Assign Compound Annotations

------------------------------------------------------------------

1. General Settings:

- Mass Tolerance: 5 ppm

2. Data Sources:

- Data Source #1: mzCloud Search

- Data Source #2: Predicted Compositions

- Data Source #3: MassList Search

- Data Source #4: ChemSpider Search

- Data Source #5: Metabolika Search

------------------------------------------------------------------

Processing node 32: Fill Gaps

------------------------------------------------------------------

1. General Settings:

- Mass Tolerance: 5 ppm

- S/N Threshold: 1.5

- Use Real Peak Detection: True

------------------------------------------------------------------

Processing node 30: Normalize Areas

------------------------------------------------------------------

1. QC-based Area Correction:

- Regression Model: Cubic Spline

- Min. QC Coverage [%]: 50

- Max. QC Area RSD [%]: 30

- Max. # Files Between QC Files: 20

2. Area Normalization:

- Normalization Type: None

- Exclude Blanks: True

------------------------------------------------------------------

Processing node 28: Mark Background Compounds

------------------------------------------------------------------

1. General Settings:

- Max. Sample/Blank: 5

- Max. Blank/Sample: 0

- Hide Background: True

------------------------------------------------------------------

Processing node 29: Predict Compositions

------------------------------------------------------------------

1. Prediction Settings:

- Mass Tolerance: 5 ppm

- Min. Element Counts: C H

- Max. Element Counts: C90 H190 Br3 Cl4 N10 O18 P3 S5

- Min. RDBE: 0

- Max. RDBE: 40

- Min. H/C: 0.1

- Max. H/C: 4

- Max. # Candidates: 10

- Max. # Internal Candidates: 200

2. Pattern Matching:

- Intensity Tolerance [%]: 30

- Intensity Threshold [%]: 0.1

- S/N Threshold: 3

- Min. Spectral Fit [%]: 30

- Min. Pattern Cov. [%]: 90

- Use Dynamic Recalibration: True

3. Fragments Matching:

- Use Fragments Matching: True

- Mass Tolerance: 5 ppm

- S/N Threshold: 3

- Search Mode: By Formula or Mass

- Mass Tolerance: 5 ppm

- Max. # of results per compound: 100

- Max. # of Predicted Compositions to be searched per Compound: 3

- Result Order (for Max. # of results per compound): Order By Reference Count (DESC)

. -----------------------------------------------------------------

Processing node 34: Map to Metabolika Pathways

------------------------------------------------------------------

1.By Mass Search Settings:

- Mass Tolerance: 5 ppm

2. By Formula Search Settings:

- Max. # of Predicted Compositions to be searched per Compound: 3

3. Display Settings:

- Max. # Pathways in 'Pathways' column: 20

Processing node 17: Differential Analysis

------------------------------------------------------------------

1. General Settings:

- Log10 Transform Values: True
